# Dietary resistant starch supplementation increases gut luminal deoxycholic acid abundance in mice

**DOI:** 10.1080/19490976.2024.2315632

**Published:** 2024-02-20

**Authors:** Melanie A. Reuter, Madelynn Tucker, Zara Marfori, Rahaf Shishani, Jessica Miranda Bustamante, Rosalinda Moreno, Michael L. Goodson, Allison Ehrlich, Ameer Y. Taha, Pamela J. Lein, Nikhil Joshi, Ilana Brito, Blythe Durbin-Johnson, Renu Nandakumar, Bethany P. Cummings

**Affiliations:** a Department of Surgery, Center for Alimentary and Metabolic Sciences, School of Medicine, University of California – Davis, Sacramento, CA, USA; b Department of Molecular Biosciences, School of Veterinary Medicine, University of California – Davis, Davis, CA, USA; c Department of Environmental Toxicology, College of Agricultural and Environmental Sciences, University of California – Davis, Davis, CA, USA; d Department of Food Science and Technology, University of California - Davis, Davis, CA, USA; e Bioinformatics Core, UC Davis Genome Center, University of California – Davis, Davis, CA, USA; f Meinig School of Biomedical Engineering, Cornell University, Ithaca, NY, USA; g Biomarkers Core Laboratory, Irving Institute for Clinical and Translational Research, Columbia University Irving Medical Center, New York, NY, USA

**Keywords:** Resistant starch, 7-α-dehydroxylation, bile acid, gut microbiome, DCA, metagenomics

## Abstract

Bile acids (BA) are among the most abundant metabolites produced by the gut microbiome. Primary BAs produced in the liver are converted by gut bacterial 7-α-dehydroxylation into secondary BAs, which can differentially regulate host health via signaling based on their varying affinity for BA receptors. Despite the importance of secondary BAs in host health, the regulation of 7-α-dehydroxylation and the role of diet in modulating this process is incompletely defined. Understanding this process could lead to dietary guidelines that beneficially shift BA metabolism. Dietary fiber regulates gut microbial composition and metabolite production. We tested the hypothesis that feeding mice a diet rich in a fermentable dietary fiber, resistant starch (RS), would alter gut bacterial BA metabolism. Male and female wild-type mice were fed a diet supplemented with RS or an isocaloric control diet (IC). Metabolic parameters were similar between groups. RS supplementation increased gut luminal deoxycholic acid (DCA) abundance. However, gut luminal cholic acid (CA) abundance, the substrate for 7-α-dehydroxylation in DCA production, was unaltered by RS. Further, RS supplementation did not change the mRNA expression of hepatic BA producing enzymes or ileal BA transporters. Metagenomic assessment of gut bacterial composition revealed no change in the relative abundance of bacteria known to perform 7-α-dehydroxylation. *P. ginsenosidimutans* and *P. multiformis* were positively correlated with gut luminal DCA abundance and increased in response to RS supplementation. These data demonstrate that RS supplementation enriches gut luminal DCA abundance without increasing the relative abundance of bacteria known to perform 7-α-dehydroxylation.

## Introduction

The gut microbiome is a crucial regulator of host health. A key mechanism by which the gut microbiome regulates host health is through the metabolites it produces. Bile acids are among the most abundant and diverse gut microbially produced metabolites. Though bile acids are well known for their role in fat digestion, they are present in the systemic circulation and act through bile acid receptors, such as Takeda G protein-coupled receptor 5 (TGR5) and Farnesoid X receptor (FXR), to regulate metabolic homeostasis, hepatic bile acid metabolism, inflammation, and other key physiologic processes.^1,2^ Primary bile acids are synthesized in the liver from cholesterol and are conjugated with taurine or glycine before leaving the liver. The most abundant primary bile acid in humans is cholic acid (CA), followed by chenodeoxycholic acid (CDCA).^3^ Once conjugated with taurine or glycine, they are stored in the gallbladder and secreted into the duodenum in response to feeding. Primary bile acids are converted into secondary bile acids through interactions with gut bacteria in the distal gastrointestinal tract. Specifically, gut bacteria convert CA into deoxycholic acid (DCA) and CDCA into lithocholic acid (LCA). Due to highly efficient gut transporters, approximately 95% of bile acids remain in the enterohepatic circulation. Some escape into the systemic circulation.^4^

Regulating bile acid production is essential for host-microbiome crosstalk. Gut microbes perform numerous modifications of luminal bile acids, which regulate host health by determining downstream signaling, as bile acid subtypes vary in their affinity for bile acid receptors. Indeed, alterations in bile acid profile are associated with many disease states, from atherosclerosis to Alzheimer’s Disease.^5,6^ The therapeutic potential of targeting the gut microbiome is highlighted by the successful application of fecal microbiota transplantation (FMT) for treating *C. difficile* infection. Notably, increases in gut bacterial bile acid metabolism have been highlighted as a critical mechanism by which FMT treats *C. difficile* infection.^7–9^ Therefore, there is untapped therapeutic potential in altering endogenous bile acid metabolism in treating and preventing various disease processes.

The pathway of converting primary bile acids to secondary bile acids, 7-α-dehydroxylation, is carried out by a series of enzymes in certain bacteria as part of a survival mechanism to detoxify their environment. As an essential pathway in bile acid metabolism, 7-α-dehydroxylation is a potential target for ameliorating disease states. Bile salt hydrolase (BSH) executes an essential first step in gut bacterial bile acid metabolism because 7-α-dehydroxylation can only begin once the bile acid is deconjugated. The first enzyme in the 7-α-dehydroxylation pathway is encoded by *baiG* and is a highly selective transporter for unconjugated bile acids.^10,11^ Unlike bacterial bile acid deconjugation, which is executed by BSH and is widely expressed across many bacterial genera in the gut microbiome, 7-α-dehydroxylation is thought to be carried out by a limited number of bacteria.^12^ It is estimated that < 1% of bacteria can perform 7-α-dehydroxylation.^13^ The most well-known players in 7-α-dehydroxylation come from the Clostridium genus.^14^ However, recent evidence has shown that other bacteria from other genera may be involved in complementary pathways.^15,16^ Therefore, our understanding of the bacteria involved and the regulation of 7-α-dehydroxylation is incomplete.^5^

In particular, our understanding of the effect of diet on gut bacterial bile acid metabolism is insufficient. Dietary fiber is a crucial regulator of gut microbial composition and metabolite production. High amylose maize resistant starch (RS) is a type two fermentable fiber that alters the bacterial microenvironment and gut bacterial metabolism.^17^ While RS is comprised of sugars that cannot be digested by human enzymes, RS can be fermented by bacteria. There is a preference for some bacteria over others to consume certain resistant starches, which promotes shifts in gut microbial composition to favor bacteria more adept at using RS as a food source.^17,18^ Dietary RS supplementation has a well-established effect to alter gut microbial metabolite production, particularly short-chain fatty acids in mice and humans.^19,20^ Interestingly, other types of dietary fiber have recently been shown to promote certain aspects of gut bacterial bile acid metabolism. Specifically, Arifuzzaman et al. reported increases in unconjugated CA, CDCA, UDCA, and MCA abundances after dietary inulin supplementation in mice, suggesting a promotion of bile acid deconjugation.^21^ They also demonstrated that bacteria mediated the change in bile acid metabolism. Oligofructose, another dietary fiber, was also found to change the bile acid pool by enhancing gut bacterial 6α-hydroxylation.^22^ However, the effect of dietary fiber on 7-α-dehydroxylation and the impact of type two dietary RS on gut bacterial bile acid metabolism has not been tested. Therefore, we tested the hypothesis that feeding mice a diet supplemented with RS would alter the gut microbiome to favor 7-α-dehydroxylation.

Herein, we report that dietary supplementation with RS enhances gut luminal DCA abundance in mice. RS did not alter CA abundance, mRNA expression of hepatic bile acid producing enzymes, or ileal bile acid transporters. Further, the effect of RS to increase gut luminal DCA production also occurred in the absence of increases in bacteria known to produce DCA. Together, these data point to a key role for dietary fiber in regulating DCA production and suggest that dietary RS supplementation increases gut bacterial DCA production through non-canonical bacteria.

## Results

### RS supplementation increased gut luminal DCA abundance

**Figure 1.**
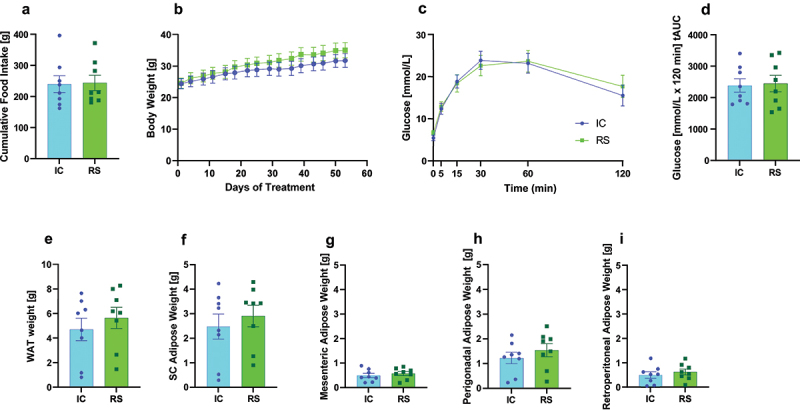
Measures of metabolic health were unchanged by RS supplementation. a) cumulative food intake (day 1–50 of IC or RS intervention), b) body weight, c) oral glucose tolerance test, d) the total area under the curve from each oral glucose tolerance test, e) total white adipose weight, f) subcutaneous adipose tissue weight, g) mesenteric adipose tissue weight, h) perigonadal adipose tissue weight, and i) retroperitoneal adipose tissue weight. Data presented as mean ± SEM, *n* = 8 per group.

To determine the impact of RS on gut bacterial bile acid metabolism, we fed male and female wild-type C57BL/6J mice a diet supplemented with RS. To control for the effect of caloric dilution by RS supplementation, we included an isocaloric (IC) control diet. The IC diet was made using a combination of amylopectin and cellulose, as previously described.^23,24^ Besides making these diets isocaloric, the percent of energy from carbohydrates was equivalent between diets. All mice began receiving the IC diet at two months of age. At four months of age, mice either continued for an additional one or two months of IC or RS intervention. Then, mice were euthanized for tissue collection. The main figures present the data from mice studied after two months of dietary intervention (six months of age). Results from mice studied after one month of dietary intervention (five months of age) are presented in the supplemental figures. We took biweekly measures of body weight and food intake for the duration of dietary intervention. On the morning of euthanasia, we performed an oral glucose tolerance test (OGTT). There were no differences in food intake, body weight, adiposity, or glucose tolerance in RS compared with IC-fed mice studied after one or two months of dietary intervention (Figure 1a–i; Supplemental Figure S1A-I). These data suggest that when controls for caloric content are in place, RS altered neither adiposity nor overall body weight. Therefore, the impact of RS reported throughout these studies is independent of food intake and body weight.

Cecal contents and serum were collected for bile acid profiling at the conclusion of the study. RS supplementation decreased total cecal bile acid concentrations compared with IC-fed mice after one month of intervention (Supplemental Table S1, *p* < .01) and tended to decrease total cecal bile acid concentrations after two months of dietary intervention, but this did not reach significance (Supplemental Table S1, *p*=.07). This finding is consistent with previous work reporting increased bile acid excretion after three weeks of fiber intervention.^21,25^ Because of the reduction in total bile acids caused by RS supplementation, we assessed both absolute bile acid concentrations and the relative proportion of specific bile acids within the total bile acid pool. Cecal bile acid concentrations as an absolute value are presented in Supplemental Table S1. The relative abundance of gut luminal bile acid concentrations normalized to total bile acid concentrations are presented in Supplemental Table S2.

**Figure 2.**
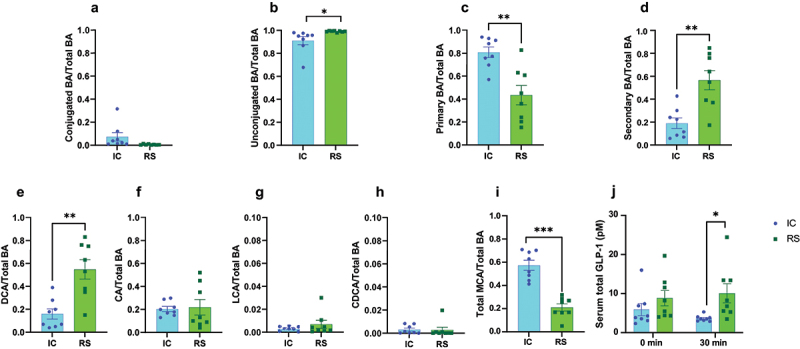
RS supplementation enhanced gut luminal DCA abundance. a) conjugated bile acids, b) unconjugated bile acids, c) primary bile acids, d) secondary bile acids, e) DCA, f) CA, g), LCA, h) CDCA, i) and total MCA levels expressed as a proportion of total bile acids in cecal contents collected from mice receiving IC or RS diet for 2 months. Data presented as mean ± SEM, *n* = 8 per group. *p<.05, **p<.01, ***p<.001 by Student’s t-test. j) circulating total GLP-1 concentrations at baseline and 30 minutes post-gavage during the OGTT. *p* < .05 by 2-factor ANOVA with Bonferroni posttest. Data presented as mean ± SEM, *n* = 8 per group.

To assess the potential impact of RS on gut bacterial bile acid metabolism, we first determined the impact of RS supplementation on unconjugated and secondary bile acid levels. After two months of dietary intervention, RS tended to decrease gut luminal conjugated bile acids and increased unconjugated bile acids as a proportion of total bile acids compared to IC-fed mice. However, this effect did not reach significance after one month of RS supplementation (Figure 2a,b, *p* < .05; Supplemental Figure 2a,b), suggesting RS favors bile acid deconjugation after prolonged RS supplementation. To evaluate 7-α-dehydroxylation, we examined the proportion of primary and secondary bile acids. Gut luminal primary bile acids as a proportion of total bile acids decreased compared to IC-fed mice after two months of dietary intervention (Figure 2c, *p* < .01). In contrast, secondary bile acids as a proportion of total bile acids increased in RS compared to IC-fed mice (Figure 2d, *p* < .01). Similar trends were observed after one month of RS supplementation, but this did not reach significance (Supplemental Figure S2C-D). The effect of RS to increase secondary bile acid abundance was primarily driven by an increase in DCA abundance. Specifically, gut luminal DCA as a proportion of total cecal bile acids tended to increase after one month of dietary intervention (Supplemental Figure 2e, *p*=.08) and increased more than three-fold compared to IC-fed mice after two months of dietary intervention (Figure 2e, *p* < .01). Additionally, glycine-conjugated DCA proportionally increased in RS compared to IC-fed mice after two months of dietary intervention, likely due to increased DCA availability as a substrate (Supplemental Table S2, *p* < .05).

CA is the substrate for gut bacterial 7-α-dehydroxylation in the production of DCA. Gut luminal CA concentrations were reduced by RS supplementation compared to IC-fed mice after one month of dietary intervention (Supplemental Table S1, *p* < .05) and tended to be reduced by RS supplementation after two months of dietary intervention (Supplemental Table S2, *p*=.08). Furthermore, gut luminal CA abundance did not differ between groups after one and two months of dietary intervention (Figure 2f; Supplemental Figure S2F), suggesting that the effect of RS to increase gut luminal DCA abundance was not driven by an increase in its substrate. Further, while RS has bile acid binding properties, when compared to bile acid binding resins, the rate is much lower.^26–29^ RS increases fecal excretion of DCA to a greater extent than CA and CDCA which are excreted to the same extent.^26,29^ Therefore, the effect of RS to increase gut luminal abundance of DCA does not appear to be driven by preferential DCA retention.

In contrast, RS did not alter gut luminal LCA relative abundance compared to IC-fed mice after one or two months of dietary intervention (Figure 2g; Supplemental Figure S2G). These data suggest RS supplementation promotes an environment that favors bacteria that selectively upregulate DCA production. While there are gut bacterial species and 7-α-dehydroxylation enzymes that can use both CA and CDCA as a substrate in the generation of secondary bile acids, there are gut bacterial species and 7-α-dehydroxylation enzymes that can only interact with CA.^14,30,31^ This demonstrates important points of divergence in 7-α-dehydroxylation between CA and CDCA. CDCA is the substrate for the production of LCA. RS supplementation decreased CDCA relative abundance compared to IC-fed mice after one month of dietary intervention (Supplemental Figure S2H, *p* < .05), but had no impact on CDCA relative abundance after two months of dietary intervention (Figure 2h). CDCA is also the substrate for hepatic muricholic acid (MCA) production. The relative abundance of gut luminal MCA was unchanged after one month of dietary intervention (Supplemental Figure S2I) and decreased after two months of RS supplementation (Figure 2i, *p* < .05).

To determine whether the effect of RS to increase gut luminal DCA abundance impacts downstream markers of bile acid activity, we measured nutrient-stimulated glucagon-like peptide-1 secretion (GLP-1). Bile acids contribute to the stimulation of GLP-1 secretion from gut enteroendocrine L cells by signaling through the bile acid receptor, TGR5.^1^ DCA is one of the strongest ligands for TGR5.^32^ Therefore, we hypothesized that the increase in gut luminal DCA abundance with RS supplementation may lead to an increase in GLP-1 secretion. Consistent with this, we find that circulating GLP-1 concentrations are increased in RS compared with IC-treated mice during the OGTT, supporting a physiologic impact for enhanced gut luminal DCA abundance (Figure 2j, *p* < .05).

### The effect of RS supplementation to increase gut luminal DCA abundance was not reflected in the circulation

**Figure 3.**
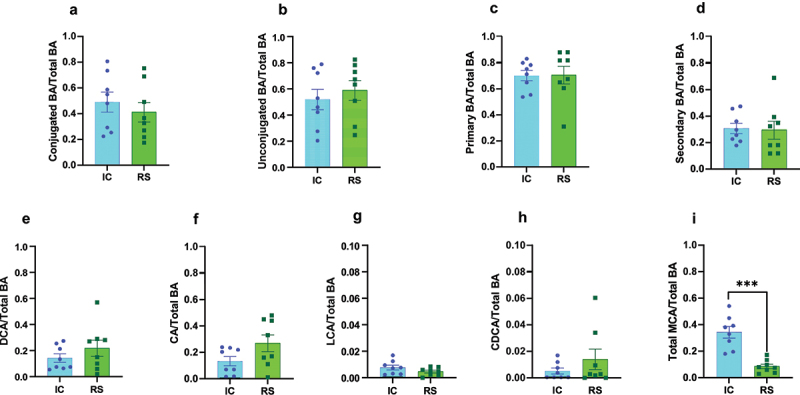
Changes in gut luminal DCA abundance were not reflected in the serum. a) conjugated bile acids, b) unconjugated bile acids, c) primary bile acids, d) secondary bile acids, e) DCA, f) CA, g) LCA, h) CDCA, and i) total MCA levels expressed as a proportion of total bile acids in fasted serum samples collected from mice receiving IC or RS diet for 2 months. *p < .05, **p < .01, ***p < .001 by Student’s t-test. Data presented as mean ± SEM, *n* = 8 per group.

In contrast to the impact of RS on bile acid concentrations in cecal contents, RS did not change the concentration of most bile acid subtypes in the circulation (Supplemental Table S3). Nevertheless, there was a decrease in circulating α-MCA, TCDCA, and TUDCA concentrations after two months of RS supplementation compared to IC-fed mice (Supplemental Table S3, *p* < .05). Further, the relative abundance of most bile acid subtypes did not differ between groups after one or two months of dietary intervention (Supplemental Table S4; Figure 3; Supplemental Figure S3). Specifically, RS did not alter the relative abundance of unconjugated bile acids or conjugated bile acids compared to IC-fed mice after two months (Figure 3a,b) but did decrease the relative abundance of conjugated bile acids with a commensurate increase in unconjugated bile acids after one month of dietary intervention (Supplemental Figure S3A-B, *p* < .05). Circulating primary and secondary bile acids as a proportion of total bile acids did not differ between groups after one or two months of RS supplementation (Supplemental Figure S3C-D; Figure 3c,d).

DCA, CA, LCA, and CDCA as a proportion of total bile acids did not differ between groups after two months of dietary intervention (Figure 3e–h). This remained mostly true in the one-month cohort (Supplemental Figure S3E, G-H), apart from circulating CA, which increased in relative abundance in RS compared to IC-fed mice (Supplemental Figure S3F, *p* < .0001). Similar to the effect of RS supplementation to decrease gut luminal MCA abundance, RS supplementation decreased circulating MCA abundance compared to IC-fed mice after one and two months of dietary intervention (Figure 3i, *p* < .01). All absolute and proportionate serum bile acid data are available in Supplemental Table S3 and Supplemental Table S4, respectively. DCA is hydrophobic and, therefore, may be preferentially sequestered into tissue. That may account for the change in cecal DCA not being reflected in serum. On the other hand, MCA is among the most hydrophilic of the bile acids and would easily partition into the blood, which may be why reductions in MCA abundance were detected in both gut luminal contents and serum.

### RS supplementation did not impact markers of hepatic bile acid metabolism or gut transporters

**Figure 4.**
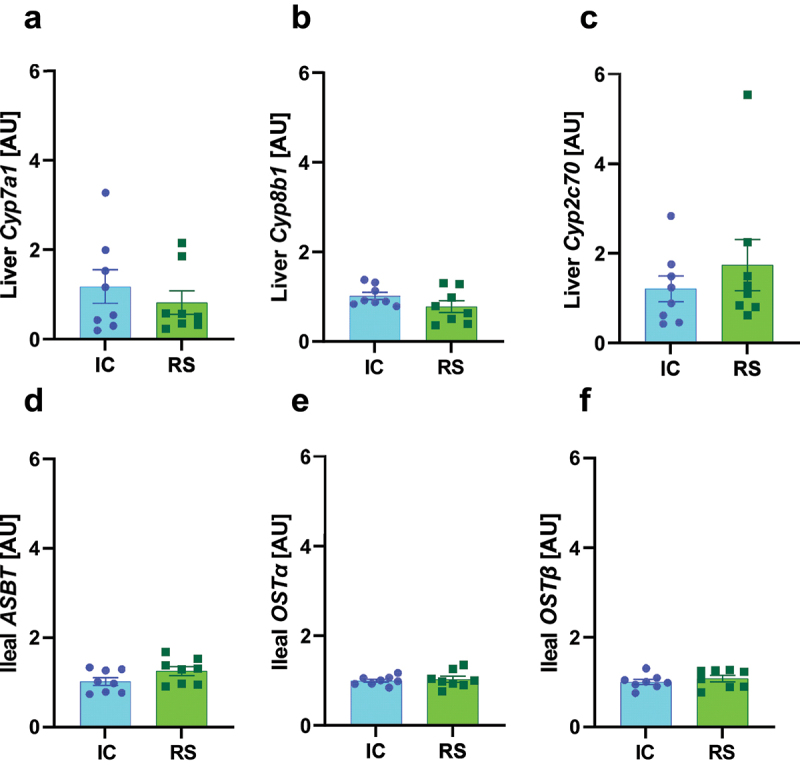
mRNA expression of hepatic bile acid producing enzymes and ileal bile acid transporters were not changed by RS supplementation. a) *Cyp7a1*, b) *Cyp8b1* and c) *Cyp2c70* mRNA expression in liver collected after 2 months of IC or RS feeding. d) *Asbt*, e) *Ost*α, and f) *Ost*β mRNA expression in ileum collected after 2 months of IC or RS feeding. Data presented as mean ± SEM, *n* = 8 per group.

To determine if alterations in hepatic bile acid metabolism or gut bile acid transport may play a role in the effect of RS to alter the bile acid profile in cecal contents, we assessed the impact of RS on the expression of hepatic bile acid metabolic enzymes and gut bile acid transporters. *Cyp7a1* is the rate-limiting step in hepatic bile acid metabolism. *Cyp8b1* is required for CA production.^33^ *Cyp7a1* and *Cyp8b1* mRNA expression did not differ between groups after one or two months of dietary intervention (Figure 4a,b; Supplemental Figure S4A-B). *Cyp2c70* is the enzyme responsible for the conversion of CDCA to MCA in mice.^34^ *Cyp2c70* mRNA expression did not differ between groups after one or two months of dietary intervention (Figure 4c, Supplemental Figure S4C).

Bile acid transporters efficiently reabsorb bile acids in the ileum. Apical sodium-dependent bile transporter (ASBT) is responsible for the ileal reuptake of bile acids.^35^ Organic solute transporter with alpha and beta subunits (OST-α OST-β) also allows bile acids to cross the basolateral membrane of enterocytes.^35^ *Asbt*, *Ostα*, and *Ostβ* mRNA expression did not differ between groups after two months of dietary intervention (Figure 4d–f). *Asbt* and *Ostα* mRNA expression did not differ between groups after one month of dietary intervention (Supplemental Figure S4D-E). However, RS increased *Ostβ* mRNA expression compared to IC-fed mice after one month of dietary intervention (Supplemental Figure S4F, *p* < .05). Together, these data suggest that alterations in hepatic bile acid enzyme expression or gut bile acid transport expression do not drive the effect of RS supplementation to increase gut luminal DCA concentrations.

### RS supplementation altered gut microbiome composition without increasing bacteria that perform 7-α-dehydroxylation

**Figure 5.**
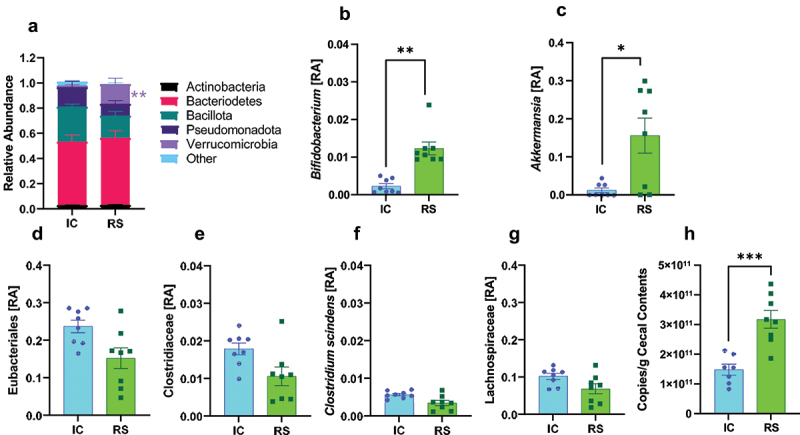
RS supplementation alters gut microbiome composition. a) phylum-level relative abundances (RA), b) *Bifidobacterium*, c) *Akkermansia* relative abundance, d) eubacteriales, e) *Clostridiaceae*, f) *Clostridium scindens*, and g) lachnospiraceae relative abundances after 2 months of IC or RS feeding. Data presented as mean ± SEM, *n* = 8 per group. *q < .05 **q < .01. h) total bacterial count from cecal contents collected after 2 months of IC or RS feeding. ***p < .001 by Student’s t-test. Data presented as mean ± SEM, *n* = 7–8 per group.

To better understand how RS alters gut luminal bile acid profile, we assessed gut microbial composition by metagenomics in the same samples used for bile acid profiling analysis. Similar to previous work, RS decreased the Shannon α-diversity index.^36,37^ Differences were considered significant with a false-discovery rate adjusted *P*-value (q-value) of less than .05. At the phylum level, RS supplementation increased the relative abundance of Verrucomicrobia compared to IC-fed mice (Figure 5a, *q* < .01). Verrucomicrobia is associated with a healthy gut microbiome but has no well-defined role in secondary bile acid production.^38^ RS supplementation increased the relative abundance of the genera *Bifidobacterium* (Figure 5b, *q <* .01) and *Akkermansia* compared to IC-fed mice (Figure 5c, *q* < .05). Both bacterial groups are associated with microbiome health, but neither has an established role in 7-α-dehydroxylation.^39,40^

We then looked closer at the impact of RS supplementation on the relative abundance of bacteria implicated in secondary bile acid production. Eubacteriales, the order that contains the Clostridium genus, tended to decrease in RS compared with IC-fed mice (Figure 5d, *q* = .11). *Clostridiaceae*, the family that includes the Clostridium genus, did not increase in response to RS supplementation (Figure 5e). *Clostridium scindens*, the predominant bacterial species known to perform 7-α-dehydroxylation,^41^ tended to decrease in RS compared to IC-fed mice (Figure 5f, *q* = .08). *Clostridium hylemonae* tended to decrease (*q* = .06) and *Peptacetobacter hiranonis* did not change. These bacteria have known 7-α-dehydroxylation capability.^42,43^ Lachnospiraceae, a family proposed to have some 7-α-dehydroxylation activity,^43^ also did not change in RS compared to IC-fed mice (Figure 5g). Several of these findings have been repeated in previous studies.^36,43,44^ To determine the impact of RS on total bacterial load, cecal contents from a separate cohort of IC and RS treated mice (treated the same as RS and IC mice used for metagenomics analysis) were studied. Total bacterial load was increased in RS compared with IC-treated mice (Figure 5h, *p* < .05).

As shown above, we found no bacteria known to perform 7-α-dehydroxylation that were increased by RS supplementation. Instead, the relative abundance of many of the bacteria known to perform 7-α-dehydroxylation tended to decrease in response to RS supplementation. Despite this, we found that RS increased gut luminal abundance of DCA without changing the amount of substrate or changing markers of hepatic bile acid metabolism or gut bile acid transport, suggesting that the effect of RS to increase gut luminal DCA abundance was mediated by the gut microbiome. In the absence of an increase in bacteria known to perform 7-α-dehydroxylation, this suggests that other players may be involved in 7-α-dehydroxylation. We assessed the correlation between bacterial abundance and gut luminal bile acid abundance to identify candidate bacteria promoting gut bacterial DCA production. The selection criteria for these bacteria were a positive association with DCA and a q-value of less than .05. We also assessed bacteria with a positive association with LCA and a q-value less than .001.

**Figure 6.**
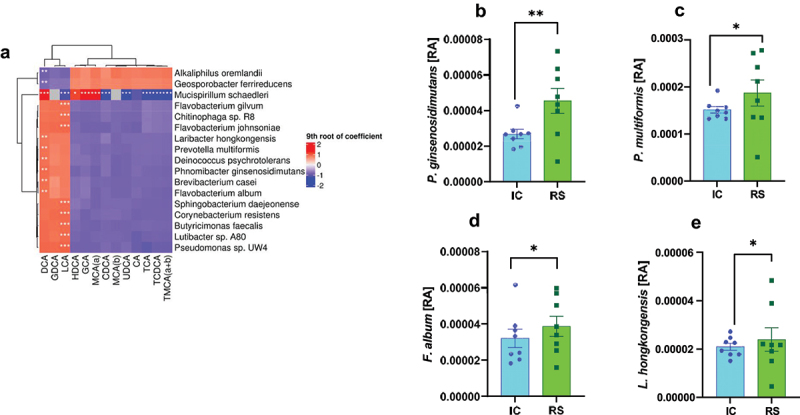
Association between bacterial relative abundance and gut luminal bile acids. A) bacterial species correlated with gut luminal bile acid abundances. Red indicates a positive correlation, and blue indicates a negative correlation. *q < .2, **q < .05, ***q < .001. b) *phnomibacter ginsenosidimutans*, c) *prevotella multiformis*, d) *flavobacterium album*, and e) *laribacter hongkongensis* relative abundances after 2 months of IC or RS feeding. *q < .05 **q < .01. Data presented as mean ± SEM, *n* = 8 per group.

The bacterium with the most positive association with DCA was *Mucispirillum schaedleri* (Figure 6a, q < .01). This bacterium was positively associated with α-MCA and GCA (q < .01). *M. schaedleri* is negatively associated with CDCA, LCA, all taurine-conjugated BAs, and UDCA. The subsequent most positively associated bacterium with DCA was *Phnomibacter ginsenosidimutans* (Figure 6a, q < .05). This bacterium was enriched in RS compared to IC-fed mice (Figure 6b, *q* < .01). Other bacteria positively associated with DCA included: *Brevibacterium casei* (q < .01), *Deinococcus psychrotolerans*, *Prevotella multiformis*, *Laribacter hongkongensis*, and *Flavobacterium album* (Figure 6a, q < .05). Of those, *P. multiformis*, *F. album*, and *L. hongkongensis* were enriched with RS compared to IC-fed mice (Figure 6c–e, q < .05). Bacteria negatively associated with DCA production were *Alkaliphilus oremlandii* and *Geosporobacter ferrireducens* (Figure 6a, q < .05). Of note, the bacteria positively associated with DCA did not overlap with those positively associated with LCA (Figure 6a). Additionally, except for *M. schaedleri*, any bacteria positively associated with DCA or LCA tended to be negatively associated with the primary bile acids. This dichotomy is consistent with a role for these bacteria in 7-α-dehydroxylation, as an increase in secondary bile acid production would reduce primary bile acid levels by shunting them into secondary bile acids.

## Discussion

Herein, we identify a new effect of dietary RS supplementation to alter gut luminal bile acid profile. Specifically, upon RS supplementation, gut luminal DCA levels were enhanced as a proportion of total bile acids. The effect of RS to increase gut luminal DCA abundance was associated with an increase in GLP-1 secretion, supporting a physiologic impact for enhanced gut luminal DCA abundance. There was no equivalent enhancement in CA, the substrate for DCA. Concurrent with our findings, resistant starch derived from lotus seeds, a type three resistant starch, has been shown to alter bile acid composition in rats.^45^ Additionally, RS did not change the mRNA expression of *Cyp7a1*, *Cyp8b1*, or *Cyp2c70* enzymes involved in cholesterol metabolism into primary bile acids. Further, RS did not alter the mRNA expression of gut bile acid transporters, *Abst*, *Ostα*, or *Ostβ*. Interestingly, LCA abundance was unchanged in response to RS supplementation. These data indicate that substrate-specific 7-α-dehydroxylation may occur in response to RS supplementation. RS caused changes in each level of bacterial taxa with no change in most known bacteria capable of 7-α-dehydroxylation. These data suggest that the effect of RS to increase DCA abundance is through promotion of gut bacterial 7-α-dehydroxylation through non-canonical bacteria.

Understanding the regulation of gut bacterial bile acid metabolism will enable the development of targeted approaches that leverage the gut microbiome to treat disease. Targeting the bacteria in the gut is attractive because it presents a minimally invasive approach with low probability of negative side-effects. For example, upregulating the 7-α-dehydroxylation pathway could increase the proportion of secondary bile acids to improve or reverse dysbiosis, as is the case with *C. difficile* infection, which is inhibited by secondary bile acids.^46^ However, efficient targeting would require detailed knowledge of the bacterial players in the pathway, the genes involved, and the enzymes at work. Though 7-α-dehydroxylation has been studied for decades and is relatively well-defined, there is still much to learn.

The 7-α-dehydroxylation pathway has been attributed to a set of genes within the bile acid inducible (bai) operon. The bai operon has been primarily characterized within *C. scindens*.^30^ The canonical bai operon includes genes *baiA* through *baiI*.^30^ Recently, more genes that could be part of the bai operon have been proposed, including the genes *baiJKL* through *baiN*.^41^ As our understanding of the bai operon evolves, so does our understanding of the bacteria capable of expressing these genes. Initially, it was believed that all 7-α-dehydroxylation was done only by *C. scindens* and, to a lesser extent, by *C. hylemonae* and *P. hiranonis*. However, other bacteria are being discovered that can perform 7-α-dehydroxylation, though there are still not many.^16,43^ Further, the microbiome is a complex system of organisms influencing each other. Other bacteria could help to upregulate 7-α-dehydroxylation without being directly capable of executing 7-α-dehydroxylation. For example, Funabashi et al. transfected *C. sporogenes*, a bacterium incapable of 7-α-dehydroxylation, with the *bai* operon.^47^ After transfection, the bacteria could convert CA to DCA, albeit with lower efficiency than those naturally possessing the *bai* operon. This work suggested that some mechanism beyond what the bai operon expresses regulates 7-α-dehydroxylation. The authors postulated that additional genes are needed to pair the pathway to enzyme cofactors from the electron transport chain.^47^ The uncertainty of which extra genes are needed and the diversity of isoforms means that identifying bacteria capable of 7-α-dehydroxylation is not as simple as screening for the *bai* operon.

Moreover, some bacterial modulation of bile acids can be influenced by genes outside of the bai operon. Epimerization and dehydrogenation are molecular rearrangements that can influence the characteristics of bile acids. These are carried out by hydroxysteroid dehydrogenases (HSDH).^48^ Epimerization has been shown to occur at all hydroxyl positions in CA and CDCA and contributes to the great diversity of bile acids.^49^ Microbes that can carry out these epimerization reactions do not need to have any *bai* genes, though many of the bacteria that do 7-α-dehydroxylation also have HSDH, such as *C. scindens*.^49,50^ Though this pathway exists outside of 7-α-dehydroxylation, these enzymes can influence 7-α-dehydroxylation by modulating bile acids to allow them to enter the pathway.^50^ Therefore, there is an avenue for indirect influence of other bacteria on 7-α-dehydroxylation, even if they don’t have the *bai* gene.

We found that RS supplementation selectively increased the production of DCA. Most of the research in the field has focused on DCA production, possibly due to the higher physiological concentrations of DCA compared to LCA.^3^ Though the process to produce both LCA and DCA has the same name, the journey is not necessarily the same for each molecule. Some enzymes have equal affinity for CA and CDCA-based intermediates. Some prefer CA species over CDCA species.^14,31^ LCA production largely depends on bacterial strain.^14^ More studies are needed to parse out differences between these pathways. Our study is not the first to show that changes in the microbiome are associated with selective 7-α-dehydroxylation. For example, Yang et al. showed that arsenic-induced dysbiosis was associated with upregulated LCA production.^51^ These data emphasize the possibility that the production of DCA and LCA is driven by distinct pathways carried out by different bacteria. Identifying experimental probes to deepen our understanding of the pathways is necessary. We used RS as a probe to manipulate bacterial abundances and bile acid metabolism. Further emphasizing this divergence, the bacteria positively associated with DCA tended to be negatively associated with LCA. Similarly, the bacteria positively associated with DCA or LCA tended to be negatively associated with all primary bile acids and their conjugated forms. This observation suggests that additional bacteria may play an important role in 7-α-dehydroxylation, though the precise mechanism is unclear. Understanding how to harness each pathway individually is essential for clinically targeting the microbiome, as abnormal ratios of LCA and DCA can be pathogenic.^52^

RS was able to change the makeup of the microbiome and selectively impact DCA production. Increased abundance of *Bifidobacterium* and *Akkermansia*, bacteria associated with a healthy microbiome, demonstrate RS’s power as a diet. Surprisingly, RS did not increase the relative abundance of bacteria known to perform 7-α-dehydroxylation, which is counterintuitive to our finding of enhanced DCA abundance in the gut lumen. Following this finding, we analyzed which bacteria correlated with DCA or LCA abundance. The species of bacteria most positively associated with a higher abundance of DCA are *M. schaedleri, Brevibacterium casei*, *Deinococcus psychrotolerans*, *Prevotella multiformis*, *Laribacter hongkongensis, Flavobacterium album*, and *P. ginsenosidimutans*.

*M. schaedleri* inhabits a particularly inhospitable region of the gut lumen, the mucosal layer. It has been implicated in inflammatory bowel disease and causally linked to Crohn’s Disease in immunodeficient mice.^53^ It tends to thrive in conditions of gut inflammation. However, it is also protective against *Salmonella* infection in mice.^53^ *B. casei* is a bacterium commonly associated with the human skin microbiome. In immunocompromised individuals, it can be pathogenic, causing systemic infection.^54^ *D. psychrotolerans* was newly discovered in 2019, and its activity is still being assessed.^55^ *P. multiformis* is a common bacterium in the oral microbiome. It is known to be inhibited by bile.^56^ This genus is associated with high fiber diets.^57^ *L. hongkongensis* is an emerging food pathogen responsible for some traveler’s diarrhea.^58^ *Flavobacterium album* was newly discovered in 2018 and its activity has not been fully determined.^59^ *P. ginsenosidimutans* was newly discovered in 2021, and its activity is still being assessed.^60^ The role of these bacteria in bile acid metabolism is incompletely understood.

Techniques for therapeutically targeting the gut microbiome are ever-evolving. While dietary intervention is one technique, another is FMT. The criteria for donation are related to the donor’s characteristics, i.e., absence of illness.^61^ However, recent research has shown that this approach needs to be revised. Successful FMT depends more on the recipient’s characteristics, i.e., which specific bacteria are over or under-abundant.^9,62^ Except in the FDA-approved case of treating antibiotic-resistant *C. difficile* infection, FMT has had mixed success as a therapeutic, especially without additional dietary intervention.^63–65^ Even so, the therapeutic profile of FMT is encouraging while the field is still determining donation best practices. Studies like this one aid in the development of a rationally designed FMT. With that approach, FMT would be based on the specific dysbiosis of the recipient, whether bacterially defined or defined by metabolites. The therapeutic potential for rationally designed FMT could increase considerably compared to the current donor-design model. However, the field needs a deeper understanding of the role of each bacterium in the microbiome and the metabolites they produce to design FMT rationally. Bile acids are the most abundant metabolites produced by gut bacteria. They enter the systemic circulation and signal to receptors throughout the body, altering gene expression, modulating signaling pathways, and more. Clinically, bile acid profile or activity downstream of bile acids are some biomarkers of successful FMT.^8,9^ It stands to reason that this pathway should be among the first to be completely defined.

We have found that RS supplementation increased DCA as a proportion of total bile acids. The effect of RS supplementation to enrich DCA was independent of CA levels, hepatic bile acid enzyme expression, and gut bile acid transporter expression. Thus, our data suggest that RS increases DCA levels through an increase in gut bacterial 7-α-dehydroxylation. However, there are limitations to our study, and further work is needed to understand the interaction between dietary resistant starch and bile acid metabolism. To control for the effect of caloric density, the IC control diet was supplemented with a non-fermentable fiber, cellulose. We did not assess bile acid levels in fecal samples and, therefore, do not know the contribution of increased bile acid excretion. However, prior work reports that RS increases fecal excretion of CA and CDCA to the same extent, and further increases DCA excretion to a greater extent.^26,29^ Therefore, the effect of RS to increase gut luminal abundance of DCA is not likely to be driven by preferential DCA retention. Previous metabolomic analysis of mouse feces and cecal contents showed that bile acids, as well as most other metabolites, are similar in each sample type, meaning the concentration of bile acids in the cecal contents should be similar to those in the feces.^66^ However, because we did not measure bile acids in the feces, we cannot determine if this is the case in our study. While we did not find an effect of RS supplementation on the relative abundance of bacteria known to perform 7-α-dehydroxylation, we did find that RS supplementation increases total bacterial load. Therefore, it is possible that the number of bacteria known to perform 7-α-dehydroxylation may still be elevated by RS in the absence of an increase in relative abundance. Further, bacterial abundance does not necessarily tell us the level of activity of a metabolic pathway of interest. Further work is needed to understand the impact of RS on expression of genes involved in gut bacteria bile acid metabolism. Overall, a deeper understanding of the regulation of 7-α-dehydroxylation could improve how we target the gut microbiome to improve host health.

## Methods

### Animals and diet

All experiments were performed in accordance with the Guide for the Care and Use of Laboratory Animals of the National Institutes of Health. The experimental protocols were approved by the Institutional Animal Care and Use Committee of the University of California at Davis. Study mice were individually housed in a temperature and humidity-controlled room with a 12:12 h light-dark cycle. At two months of age, male and female C57BL/6J mice were put on an isocaloric diet (IC) (Research Diets Inc; diet number D21102808M) for two months. Diet composition is available in Supplemental Table S5. The IC diet was 12.3% of energy protein, 45.8% of energy carbohydrate, and 40.1% of energy fat, totaling 4085 kcal/kg. Upon enrollment, mice continued to be fed the IC diet or a diet supplemented with RS (Research Diets Inc; diet number D21102809M) for 1 or 2 more months. Groups were age, sex, and weight-matched at the time of enrollment. The RS diet was 12.3% protein, 45.8% carbohydrate, and 40.1% fat of total kcal with 4085 kcal/kg. Both diets were equivalently enriched for fat (24.7 g/kg butter, 61.9 g/kg beef tallow, and 95.3 g/kg soybean oil in both diets) to induce metabolic stress. We included an IC-fed control group to control the effect of caloric dilution by RS supplementation. The IC diet was made using a combination of amylopectin and cellulose, as previously described.^23,24^ The IC diet was supplemented with cellulose, while the RS diet used the fermentable starch, high-amylose maize (Hi-maize 260®). Diet was analyzed and validated by Eurofins Nutrition Analysis Center, Madison, WI, US assay AOAC 2002.02 2005, validating that 16.5% of the RS diet contains RS and that RS levels in the IC were undetectable. Eurofins Nutrition Analysis assay CFR-21 calc validated that the energy from carbohydrates was equivalent between diets. Body weight and food intake measurements were collected twice per week.

Mice underwent an oral glucose tolerance test (OGTT, 4 mL/kg body weight oral gavage with a dextrose dose of 2 g/kg) on the day of euthanasia (either 4 weeks or 8 weeks after the start of dietary intervention). Glucose was measured at 0, 5, 15, 30, 60, and 120 minutes after oral glucose gavage using a OneTouch Ultra2 Blood Glucose Monitoring System glucometer (Life Scan Inc, Milpitas, CA, USA). Serum for bile acid quantification was collected before the administration of glucose. Serum collected during the OGTT was used to measure total GLP-1 with the U-PLEX Metabolic Group 1 Multiplex Assay (MesoScale Diagnostics, Rockville, Maryland, USA). Following OGTT, mice were euthanized with an overdose of pentobarbital (200 mg/kg IP), and tissues were collected and weighed. Cecal contents for bile acid quantification were collected at this time. Serum and cecal contents were frozen immediately and stored at −80°C until they were thawed for use. Tissues collected included the median lobe of the liver and the ileum, which were used for qPCR. This study examined two cohorts: those on diet intervention for one month and those on diet intervention for two months.

### Bile acid quantification

Serum and cecal contents were analyzed for bile acids by UPLC-MS/MS at the Biomarkers Core Laboratory in the Irving Institute for Clinical and Translational Research at Columbia University Medical Center as previously described using Waters Xevo TQS mass spectrometer integrated with an Acquity UPLC system (Milford, MA).^67^ The lower limits of quantitation (LOQ) for the bile acids was 1 nM, defined as the lowest concentration with an accuracy and precision of < 20%. Intra-assay precision for the measured bile acids ranged from 1.49%-5.8% while the inter-assay precision ranged from 2.9% to 5.07%.

### qPCR

qPCR was performed as previously described.^68^ To extract the RNA, liver tissue samples were disrupted and homogenized using in-house buffers. A solution of 4 M Guanidine thiocyanate, 10 mM MES at a pH of 5.5, and 1% β-mercaptoethanol was added in a volume of 600 μL to a 2 mL tube containing liver tissue and ceramic beads (Qiagen 13,114–325). The solution was homogenized using a PRECELLYS® Evolution super homogenizer (Bertin Technologies, Montigny de Bretonneux, France; Serial no: 300 2319) at 550 rpm for 20 seconds, followed by a 30-second rest. The resulting solution was centrifuged at 8000 rcf for three minutes. The supernatant was collected, 350 µL of 70% ethanol was added, placed into a spin column (Epoch Sciences 1920–05), and centrifuged at 8000 rcf for 30 seconds. A solution of 1 M guanidine thiocyanate and 10 mM of Tris-HCL at a pH of 7.0 was added to the spin column. Again, this solution was centrifuged at 8000 rcf for 30 seconds. A solution of 80% ethanol and 10 mM of Tris-HCL at a pH of 7.0 was added and centrifuged at 8000 rcf for 30 seconds twice. This final solution that remained in the spin column was placed in a new tube and centrifuged at 8000 rcf for two minutes. The sample was washed with RNase-free water and centrifuged at 8000 rcf for 30 seconds. Purity and concentration were assessed using a NanoDrop One (Thermo Fisher Scientific, Madison, WI, USA; Catalog number: ND-ONE-W).

cDNA from above was added to each well of a 96-well plate. A MasterMix solution containing 10 µL of Sso Advanced Universal SYRB Green Mastermix (Bio-Rad Laboratories Inc; Catalog number: 1725272), 1 µL of both forward and reverse primer, and 3 µL of DNase-RNase Free water was added in a volume of 15 µL to each well. Primers were custom ordered from Integrated DNA Technologies (Coralville, IA, USA; Sequence in Supplemental Table S6). Plates were sealed using Excel Scientific eXTReme Sealing Films (Genesee Scientific, San Diego, CA, USA; Catalog number 12–703). Plates were spun at 25,000 rpm for one minute. The plates were read in a QuantStudio 6 Flex reader (Applied Biosystems, Waltham, MA, USA; catalog number 4,485,691). Primers are available in Supplemental Table S6.

### Metagenomics and microbial analysis

The UC Davis DNA Technologies and Expression Analysis Core processed cecal contents. The samples were DNA extracted using a zymoBIOMICS DNA/RNA miniprep kit (Zymo Research Corporation, Irvine, CA, USA). Quality control, standardization, and library preparation were done as previously reported.^65,69^ The sequencing was pair-end 2 × 150bp using the NovaSeq 6000 system (Illumina, San Diego, CA, USA) with 1% phi X sequencing control spike-in. The raw read data was filtered using HTStream (version 1.3.3), which included screening for contaminants (such as PhiX), quality-based trimming, deduplication, and adapter trimming.^70^ Bowtie2 (version 2.4.2),^71^ along with Samtools (version 1.15.1) and Bedtools2 (version 2.29.2),^72,73^ were used to remove any host contamination. Finally, metagenomics classification was performed using kraken2 (version 2.1.3),^74^ and kraken-biom (version 1.0.1) as used to convert into biom format files.^75^

Preprocessing of metagenomics data was conducted using the Bioconductor package [phyloseq], version 1.42.0.^76^ Multidimensional scaling plots used classical multidimensional scaling and were based on Bray distances of log-transformed RLE normalized species-level taxon counts.^77–79^ Shannon alpha diversity was compared between groups using a two-sample t-test. Differential abundance analyses and associations of taxa abundance with bile acid levels were conducted on RLE normalized counts using the R package Tweedieverse, version 0.0.1 with a compound Poisson linear model.^80,81^ Analyses were conducted using R version 4.2.2 (2022-10-31).^82^ Story q-value (false discovery rate adjusted p-value). A q-value less than .05 indicates statistical significance. Differential abundance analyses included taxa present in eight or more samples (distributed among diets in any way). Relative abundances were used to visually represent the data, with statistical significance being determined using the RLE normalized abundances.

### Cecal content total bacterial quantification

Total bacterial load from cecal contents from representative mice in each treatment group was quantified using the method described by Brukner, *et al*.^83^ Briefly, genomic DNA was isolated from 40–140 mg of cecal contents. The total number of bacteria were quantified by qPCR using purified *Lactococcus lactis* genomic DNA as the standard (a generous gift from the laboratory of David A. Mills at UC Davis). Primers are available in Supplemental Table S6. The total number of bacteria were normalized to the mass amount of cecal contents.

### Data analysis and statistics

Data are presented as mean ± SEM. All statistical analyses were performed using GraphPad Prism 9.3.1 for Windows (GraphPad Software, San Diego, CA, USA), except metagenomics data were analyzed as described above. Data were analyzed using an unpaired, two-tailed t-test or two-factor repeated measures ANOVA with Bonferroni posttest, as appropriate. Differences were considered significant at *p* < .05.

## Supplementary Material

Supplement.docx

## Data Availability

All metagenomics data are available at SRA.

## References

[1] Thomas C, Gioiello A, Noriega L, Strehle A, Oury J, Rizzo G, Macchiarulo A, Yamamoto H, Mataki C, Pruzanski M. et al. TGR5-mediated bile acid sensing controls glucose homeostasis. Cell Metab. 2009;10(3):167–18. doi:10.1016/j.cmet.2009.08.001. 19723493 PMC2739652

[2] Wang H, Chen J, Hollister K, Sowers LC, Forman BM. Endogenous bile acids are ligands for the nuclear receptor FXR/BAR. Mol Cell. 1999;3(5):543–553. doi:10.1016/S1097-2765(00)80348-2. 10360171

[3] Di Ciaula A, Garruti G, Lunardi Baccetto R, Molina-Molina E, Bonfrate L, Wang DQ, Portincasa P. Bile acid physiology. Ann Hepatol. 2017;16 (Suppl 1):S4–S14. doi:10.5604/01.3001.0010.5493. 29080336

[4] Chiang JY. Bile acid metabolism and signaling. Compr Physiol. 2013;3:1191–1212. 23897684 10.1002/cphy.c120023 PMC4422175

[5] Charach G, Grosskopf I, Rabinovich A, Shochat M, Weintraub M, Rabinovich P. The association of bile acid excretion and atherosclerotic coronary artery disease. Therap Adv Gastroenterol. 2011;4(2):95–101. doi:10.1177/1756283X10388682. PMC3105622 21694811

[6] MahmoudianDehkordi S, Arnold M, Nho K, Ahmad S, Jia W, Xie G, Louie G, Kueider‐Paisley A, Moseley MA, Thompson JW. et al. Altered bile acid profile associates with cognitive impairment in Alzheimer’s disease—an emerging role for gut microbiome. Alzheimer’s & Dementia. 2019;15(1):76–92. doi:10.1016/j.jalz.2018.07.217. PMC6487485 30337151

[7] Mullish BH, McDonald JAK, Pechlivanis A, Allegretti JR, Kao D, Barker GF, Kapila D, Petrof EO, Joyce SA, Gahan CGM. et al. Microbial bile salt hydrolases mediate the efficacy of faecal microbiota transplant in the treatment of recurrent Clostridioides difficile infection. Gut. 2019;68(10):1791–1800. doi:10.1136/gutjnl-2018-317842. 30816855 PMC6839797

[8] Weingarden AR, Chen C, Bobr A, Yao D, Lu Y, Nelson VM, Sadowsky MJ, Khoruts A. Microbiota transplantation restores normal fecal bile acid composition in recurrent Clostridium difficile infection. Am J Physiol Gastrointest Liver Physiol. 2014;306(4):G310–9. doi:10.1152/ajpgi.00282.2013. 24284963 PMC3920123

[9] Danne C, Rolhion N, Sokol H. Recipient factors in faecal microbiota transplantation: one stool does not fit all. Nat Rev Gastroenterol Hepatol. 2021;18(7):503–513. doi:10.1038/s41575-021-00441-5. 33907321

[10] Ridlon JM, Harris SC, Bhowmik S, Kang DJ, Hylemon PB. Consequences of bile salt biotransformations by intestinal bacteria. Gut Microbes. 2016;7(1):22–39. doi:10.1080/19490976.2015.1127483. 26939849 PMC4856454

[11] Mallonee DH, Hylemon PB. Sequencing and expression of a gene encoding a bile acid transporter from Eubacterium sp. strain VPI 12708. J Bacteriol. 1996;178(24):7053–7058. doi:10.1128/jb.178.24.7053-7058.1996. 8955384 PMC178615

[12] Song Z, Cai Y, Lao X, Wang X, Lin X, Cui Y, Kalavagunta PK, Liao J, Jin L, Shang J. et al. Taxonomic profiling and populational patterns of bacterial bile salt hydrolase (BSH) genes based on worldwide human gut microbiome. Microbiome. 2019;7(1):9. doi:10.1186/s40168-019-0628-3. 30674356 PMC6345003

[13] Rath S, Rud T, Karch A, Pieper DH, Vital M. Pathogenic functions of host microbiota. Microbiome. 2018;6(1):174. doi:10.1186/s40168-018-0542-0. 30266099 PMC6162913

[14] Hirano S, Nakama R, Tamaki M, Masuda N, Oda H. Isolation and characterization of thirteen intestinal microorganisms capable of 7 alpha-dehydroxylating bile acids. Appl Environ Microbiol. 1981;41(3):737–745. doi:10.1128/aem.41.3.737-745.1981. 7224633 PMC243769

[15] Lee JW, Cowley ES, Wolf PG, Doden HL, Murai T, Caicedo KYO, Ly LK, Sun F, Takei H, Nittono H. et al. Formation of secondary allo-bile acids by novel enzymes from gut firmicutes. Gut Microbes. 2022;14(1):2132903. doi:10.1080/19490976.2022.2132903. 36343662 PMC9645264

[16] Sato Y, Atarashi K, Plichta DR, Arai Y, Sasajima S, Kearney SM, Suda W, Takeshita K, Sasaki T, Okamoto S. et al. Novel bile acid biosynthetic pathways are enriched in the microbiome of centenarians. Nature. 2021;599(7885):458–464. doi:10.1038/s41586-021-03832-5. 34325466

[17] Martinez I, Kim J, Duffy PR, Schlegel VL, Walter J, Heimesaat MM. Resistant starches types 2 and 4 have differential effects on the composition of the fecal microbiota in human subjects. PLoS One. 2010;5(11):e15046. doi:10.1371/journal.pone.0015046. 21151493 PMC2993935

[18] Dobranowski PA, Stintzi A. Resistant starch, microbiome, and precision modulation. Gut Microbes. 2021;13(1):1926842. doi:10.1080/19490976.2021.1926842. 34275431 PMC8288039

[19] Bodinham CL, Smith L, Thomas EL, Bell JD, Swann JR, Costabile A, Russell-Jones D, Umpleby AM, Robertson MD. Efficacy of increased resistant starch consumption in human type 2 diabetes. Endocr Connect. 2014;3(2):75–84. doi:10.1530/EC-14-0036. 24671124 PMC3987287

[20] Lange K, Hugenholtz F, Jonathan MC, Schols HA, Kleerebezem M, Smidt H, Müller M, Hooiveld GJEJ. Comparison of the effects of five dietary fibers on mucosal transcriptional profiles, and luminal microbiota composition and SCFA concentrations in murine colon. Mol Nutr Food Res. 2015;59(8):1590–1602. doi:10.1002/mnfr.201400597. 25914036

[21] Arifuzzaman M, Won TH, Li TT, Yano H, Digumarthi S, Heras AF, Zhang W, Parkhurst CN, Kashyap S, Jin W-B. et al. Inulin fibre promotes microbiota-derived bile acids and type 2 inflammation. Nature. 2022;611(7936):578–584. doi:10.1038/s41586-022-05380-y. 36323778 PMC10576985

[22] Makki K, Brolin H, Petersen N, Henricsson M, Christensen DP, Khan MT, Wahlström A, Bergh P-O, Tremaroli V, Schoonjans K. et al. 6α-hydroxylated bile acids mediate TGR5 signalling to improve glucose metabolism upon dietary fiber supplementation in mice. Gut. 2023;72(2):314–324. doi:10.1136/gutjnl-2021-326541. 35697422 PMC9872241

[23] Charrier JA, Martin RJ, McCutcheon KL, Raggio AM, Goldsmith F, Goita M, Senevirathne RN, Brown IL, Pelkman C, Zhou J. et al. High fat diet partially attenuates fermentation responses in rats fed resistant starch from high-Amylose Maize. Obesity. 2013;21(11):2350–2355. doi:10.1002/oby.20362. 23512798 PMC5225625

[24] Vidrine K, Ye J, Martin RJ, McCutcheon KL, Raggio AM, Pelkman C, Durham HA, Zhou J, Senevirathne RN, Williams C. et al. Resistant starch from high amylose maize (HAM-RS2) and dietary butyrate reduce abdominal fat by a different apparent mechanism. Obesity (Silver Spring). 2014;22(2):344–348. doi:10.1002/oby.20501. 23630079

[25] van Munster IP, Tangerman A, Nagengast FM. Effect of resistant starch on colonic fermentation, bile acid metabolism, and mucosal proliferation. Dig Dis Sci. 1994;39(4):834–842. doi:10.1007/BF02087431. 8149850

[26] Abadie C, Hug M, Kubli C, Gains N. Effect of cyclodextrins and undigested starch on the loss of chenodeoxycholate in the faeces. Biochem J. 1994;299(Pt 3):725–730. doi:10.1042/bj2990725. 8192660 PMC1138080

[27] Ebihara K, Shiraishi R, Okuma K. Hydroxypropyl-modified potato starch increases fecal bile acid excretion in rats. J Nutr. 1998;128(5):848–854. doi:10.1093/jn/128.5.848. 9566992

[28] Lopez HW, Levrat-Verny MA, Coudray C, Besson C, Krespine V, Messager A, Demigné C, Rémésy C. Class 2 resistant starches lower plasma and liver lipids and improve mineral retention in rats. J Nutr. 2001;131(4):1283–1289. doi:10.1093/jn/131.4.1283. 11285339

[29] Trautwein EA, Forgbert K, Rieckhoff D, Erbersdobler HF. Impact of β-cyclodextrin and resistant starch on bile acid metabolism and fecal steroid excretion in regard to their hypolipidemic action in hamsters1Presented in part at experimental biology 98, 18–22 April, 1998, San Francisco, CA, USA.1. Biochim Biophys Acta. 1999;1437(1):1–12. doi:10.1016/S0005-2760(98)00174-X. 9931405

[30] Ridlon JM, Kang DJ, Hylemon PB. Bile salt biotransformations by human intestinal bacteria. J Lipid Res. 2006;47(2):241–259. doi:10.1194/jlr.R500013-JLR200. 16299351

[31] Ridlon JM, Hylemon PB. Identification and characterization of two bile acid coenzyme a transferases from clostridium scindens, a bile acid 7α-dehydroxylating intestinal bacterium. J Lipid Res. 2012;53(1):66–76. doi:10.1194/jlr.M020313. 22021638 PMC3243482

[32] Kawamata Y, Fujii R, Hosoya M, Harada M, Yoshida H, Miwa M, Fukusumi S, Habata Y, Itoh T, Shintani Y. et al. A G protein-coupled receptor responsive to bile acids. J Biol Chem. 2003;278(11):9435–9440. doi:10.1074/jbc.M209706200. 12524422

[33] Chiang JYL, Ferrell JM. Up to date on cholesterol 7 alpha-hydroxylase (CYP7A1) in bile acid synthesis. Liver Res. 2020;4(2):47–63. doi:10.1016/j.livres.2020.05.001. 34290896 PMC8291349

[34] Takahashi S, Fukami T, Masuo Y, Brocker CN, Xie C, Krausz KW, Wolf CR, Henderson CJ, Gonzalez FJ. Cyp2c70 is responsible for the species difference in bile acid metabolism between mice and humans. J Lipid Res. 2016;57(12):2130–2137. doi:10.1194/jlr.M071183. 27638959 PMC5321228

[35] Dawson PA, Lan T, Rao A. Bile acid transporters. J Lipid Res. 2009;50(12):2340–2357. doi:10.1194/jlr.R900012-JLR200. 19498215 PMC2781307

[36] Bendiks ZA, Guice J, Coulon D, Raggio AM, Page RC, Carvajal-Aldaz DG, Luo M, Welsh DA, Marx BD, Taylor CM. et al. Resistant starch type 2 and whole grain maize flours enrich different intestinal bacteria and metatranscriptomes. J Funct Foods. 2022;90:104982. doi:10.1016/j.jff.2022.104982.

[37] Bendiks ZA, Knudsen KEB, Keenan MJ, Marco ML. Conserved and variable responses of the gut microbiome to resistant starch type 2. Nutr Res. 2020;77:12–28. doi:10.1016/j.nutres.2020.02.009. 32251948 PMC7295659

[38] Fujio-Vejar S, Vasquez Y, Morales P, Magne F, Vera-Wolf P, Ugalde JA, Navarrete P, Gotteland M. The gut microbiota of healthy Chilean subjects reveals a high abundance of the phylum verrucomicrobia. Front Microbiol. 2017;8:1221. doi:10.3389/fmicb.2017.01221. 28713349 PMC5491548

[39] Belzer C, de Vos WM. Microbes inside—from diversity to function: the case of Akkermansia. ISME J. 2012;6(8):1449–1458. doi:10.1038/ismej.2012.6. 22437156 PMC3401025

[40] Hidalgo-Cantabrana C, Delgado S, Ruiz L, Ruas-Madiedo P, Sanchez B, Margolles A, Britton RA, Cani PD. Bifidobacteria and their health-promoting effects. Microbiol Spectr. 2017;5(3):73–98. doi:10.1128/microbiolspec.BAD-0010-2016. PMC11687494 28643627

[41] Ridlon JM, Kang DJ, Hylemon PB, Bajaj JS. Bile acids and the gut microbiome. Curr Opin Gastroenterol. 2014;30(3):332–338. doi:10.1097/MOG.0000000000000057. 24625896 PMC4215539

[42] Ridlon JM, Kang DJ, Hylemon PB. Isolation and characterization of a bile acid inducible 7α-dehydroxylating operon in clostridium hylemonae TN271. Anaerobe. 2010;16(2):137–146. doi:10.1016/j.anaerobe.2009.05.004. 19464381 PMC6262846

[43] Vital M, Rud T, Rath S, Pieper DH, Schluter D. Diversity of bacteria exhibiting bile acid-inducible 7alpha-dehydroxylation genes in the human gut. Comput Struct Biotechnol J. 2019;17:1016–1019. doi:10.1016/j.csbj.2019.07.012. 31428294 PMC6692061

[44] Sybille T, June Z, Michael K, Roy M, Maria LM. The intestinal microbiota in aged mice is modulated by dietary resistant starch and correlated with improvements in host responses. FEMS Microbiol Ecol. 2013;83(2):299–309. doi:10.1111/j.1574-6941.2012.01475.x. 22909308

[45] Lei S, He S, Li X, Zheng B, Zhang Y, Zeng H. Effect of lotus seed resistant starch on small intestinal flora and bile acids in hyperlipidemic rats. Food Chem. 2023;404:134599. doi:10.1016/j.foodchem.2022.134599. 36444019

[46] Buffie CG, Bucci V, Stein RR, McKenney PT, Ling L, Gobourne A, No D, Liu H, Kinnebrew M, Viale A. et al. Precision microbiome reconstitution restores bile acid mediated resistance to Clostridium difficile. Nature. 2015;517(7533):205–208. doi:10.1038/nature13828. 25337874 PMC4354891

[47] Funabashi M, Grove TL, Wang M, Varma Y, McFadden ME, Brown LC, Guo C, Higginbottom S, Almo SC, Fischbach MA. A metabolic pathway for bile acid dehydroxylation by the gut microbiome. Nature. 2020;582(7813):566–570. doi:10.1038/s41586-020-2396-4. 32555455 PMC7319900

[48] Larabi AB, Masson HLP, Baumler AJ. Bile acids as modulators of gut microbiota composition and function. Gut Microbes. 2023;15(1):2172671. doi:10.1080/19490976.2023.2172671. 36740850 PMC9904317

[49] Guzior DV, Quinn RA. Review: microbial transformations of human bile acids. Microbiome. 2021;9(1):140. doi:10.1186/s40168-021-01101-1. 34127070 PMC8204491

[50] Devendran S, Mendez-Garcia C, Ridlon JM. Identification and characterization of a 20β-HSDH from the anaerobic gut bacterium Butyricicoccus desmolans ATCC 43058. J Lipid Res. 2017;58(5):916–925. doi:10.1194/jlr.M074914. 28314858 PMC5408610

[51] Yang Y, Chi L, Liu CW, Hsiao YC, Lu K. Chronic Arsenic Exposure Perturbs Gut Microbiota and Bile Acid Homeostasis in Mice. Chem Res Toxicol. 2023;36(7):1037–1043. doi:10.1021/acs.chemrestox.2c00410. 37295807 PMC10773974

[52] Owen RW, Henly PJ, Thompson MH, Hill MJ. Steroids and cancer: faecal bile acid screening for early detection of cancer risk. J Steroid Biochem. 1986;24(1):391–394. doi:10.1016/0022-4731(86)90088-9. 3702422

[53] Herp S, Durai Raj AC, Salvado Silva M, Woelfel S, Stecher B. The human symbiont mucispirillum schaedleri: causality in health and disease. Med Microbiol Immunol. 2021;210(4):173–179. doi:10.1007/s00430-021-00702-9. 34021796 PMC7615636

[54] Gruner E, Steigerwalt AG, Hollis DG, Weyant RS, Weaver RE, Moss CW, Daneshvar M, Brown JM, Brenner DJ. Human infections caused by Brevibacterium casei, formerly CDC groups B-1 and B-3. J Clin Microbiol. 1994;32(6):1511–1518. doi:10.1128/jcm.32.6.1511-1518.1994. 8077397 PMC264029

[55] Tian J, Wang L, Liu P, Geng Y, Zhu G, Zheng R, Liu Z, Zhao Y, Yang J, Peng F. *Deinococcus psychrotolerans* sp. nov., isolated from soil on the South Shetland Islands, Antarctica. Int J Syst Evol Microbiol. 2019;69(12):3696–3701. doi:10.1099/ijsem.0.003484. 31647398

[56] Sakamoto M, Huang Y, Umeda M, Ishikawa I, Benno Y. *Prevotella multiformis*sp. nov., isolated from human subgingival plaque. Int J Syst Evol Microbiol. 2005;55(2):815–819. doi:10.1099/ijs.0.63451-0. 15774668

[57] Wu GD, Chen J, Hoffmann C, Bittinger K, Chen YY, Keilbaugh SA, Bewtra M, Knights D, Walters WA, Knight R. et al. Linking long-term dietary patterns with gut microbial enterotypes. Science. 2011;334(6052):105–108. doi:10.1126/science.1208344. 21885731 PMC3368382

[58] Ekundayo TC, Igere BE, Iwu CD, Oluwafemi YD, Tiamiyu AM, Adesina IA, Anuoluwa IA, Ekundayo EA, Bello OO, Olaniyi OO. et al. Prevalence of laribacter hongkongensis in food and environmental matrices: a systematic review and meta-analysis. Food Microbiol. 2022;107:104089. doi:10.1016/j.fm.2022.104089. 35953181

[59] Baek C, Shin SK, Yi H. *Flavobacterium magnum* sp. nov., *Flavobacterium pallidum* sp. nov., *Flavobacterium crocinum* sp. nov. and *Flavobacterium album* sp. nov. Int J Syst Evol Microbiol. 2018;68(12):3837–3843. doi:10.1099/ijsem.0.003067. 30320543

[60] Siddiqi MZ, Sambath P, Im WT. *Phnomibacter ginsenosidimutans* gen. nov., sp. nov., a novel glycoside hydrolase positive bacterial strain with ginsenoside hydrolysing activity. Int J Syst Evol Microbiol. 2021;71(5):71. doi:10.1099/ijsem.0.004793. 33974532

[61] Ng SC, Kamm MA, Yeoh YK, Chan PKS, Zuo T, Tang W, Sood A, Andoh A, Ohmiya N, Zhou Y. et al. Scientific frontiers in faecal microbiota transplantation: joint document of Asia-Pacific Association of Gastroenterology (APAGE) and Asia-Pacific Society for Digestive Endoscopy (APSDE). Gut. 2020;69(1):83–91. doi:10.1136/gutjnl-2019-319407. 31611298 PMC6943253

[62] Kootte RS, Levin E, Salojarvi J, Smits LP, Hartstra AV, Udayappan SD, Hermes G, Bouter KE, Koopen AM, Holst JJ. et al. Improvement of insulin sensitivity after lean donor feces in Metabolic Syndrome is Driven by baseline intestinal microbiota composition. Cell Metab. 2017;26(4):611–619.e6. doi:10.1016/j.cmet.2017.09.008. 28978426

[63] Weingarden AR, Dosa PI, DeWinter E, Steer CJ, Shaughnessy MK, Johnson JR, Khoruts A, Sadowsky MJ. Changes in colonic bile acid composition following fecal microbiota transplantation are sufficient to control clostridium difficile germination and growth. PloS One. 2016;11(1):e0147210. doi:10.1371/journal.pone.0147210. 26789728 PMC4720481

[64] Allegretti JR, Kassam Z, Mullish BH, Chiang A, Carrellas M, Hurtado J, Marchesi JR, McDonald JAK, Pechlivanis A, Barker GF. et al. Effects of fecal microbiota transplantation with oral capsules in obese patients. Clin Gastroenterol Hepatol. 2020;18(4):855–863.e2. doi:10.1016/j.cgh.2019.07.006. 31301451

[65] Allegretti JR, Kassam Z, Hurtado J, Marchesi JR, Mullish BH, Chiang A, Thompson CC, Cummings BP. Impact of fecal microbiota transplantation with capsules on the prevention of metabolic syndrome among patients with obesity. Hormones (Athens). 2021;20(1):209–211. doi:10.1007/s42000-020-00265-z. 33420959 PMC8432937

[66] Zeng H, Grapov D, Jackson MI, Fahrmann J, Fiehn O, Combs GF. Integrating multiple analytical datasets to compare metabolite profiles of mouse colonic-cecal contents and feces. Metabolites. 2015;5(3):489–501. doi:10.3390/metabo5030489. 26378591 PMC4588808

[67] Urso A, Leiva-Juarez MM, Briganti DF, Aramini B, Benvenuto L, Costa J, Nandakumar R, Gomez EA, Robbins HY, Shah L. et al. Aspiration of conjugated bile acids predicts adverse lung transplant outcomes and correlates with airway lipid and cytokine dysregulation. J Heart Lung Transplant. 2021;40(9):998–1008. doi:10.1016/j.healun.2021.05.007. 34183226 PMC9326874

[68] Holter MM, Phuong DJ, Lee I, Saikia M, Weikert L, Fountain S, Anderson ET, Fu Q, Zhang S, Sloop KW. et al. 14-3-3-zeta mediates GLP-1 receptor agonist action to alter α cell proglucagon processing. Sci Adv. 2022;8(29):eabn3773. doi:10.1126/sciadv.abn3773. 35867787 PMC9307243

[69] Bustamante JM, Dawson T, Loeffler C, Marfori Z, Marchesi JR, Mullish BH, Thompson CC, Crandall KA, Rahnavard A, Allegretti JR. et al. Impact of Fecal Microbiota Transplantation on Gut Bacterial Bile Acid Metabolism in Humans. Nutrients. 2022;14(24):14. doi:10.3390/nu14245200. PMC9785599 36558359

[70] Petersen KR, Streett DA, Gerritsen AT, Hunter SS, Settles ML. Super deduper, fast PCR duplicate detection in fastq files. Association for Computing Machinery Conference on Bioinformatics, Computational Biology, and Health Informatics; 2015;491–492. Atlanta, GA.

[71] Langmead B, Salzberg SL. Fast gapped-read alignment with bowtie 2. Nat Methods. 2012;9(4):357–359. doi:10.1038/nmeth.1923. 22388286 PMC3322381

[72] Li H, Handsaker B, Wysoker A, Fennell T, Ruan J, Homer N, Marth G, Abecasis G, Durbin R. The sequence Alignment/Map format and SAMtools. Bioinformatics. 2009;25(16):2078–2079. doi:10.1093/bioinformatics/btp352. 19505943 PMC2723002

[73] Quinlan AR, Hall IM. Bedtools: a flexible suite of utilities for comparing genomic features. Bioinformatics. 2010;26(6):841–842. doi:10.1093/bioinformatics/btq033. 20110278 PMC2832824

[74] Wood DE, Lu J, Langmead B. Improved metagenomic analysis with Kraken 2. Genome Biol. 2019;20(1):257. doi:10.1186/s13059-019-1891-0. 31779668 PMC6883579

[75] Dabdoub S. kraken-biom: Enabling interoperative format conversion for Kraken results (Version 1.2) [Software]. 2016.

[76] McMurdie PJ, Holmes S, Watson M. Phyloseq: an R package for reproducible interactive analysis and graphics of microbiome census data. PLoS One. 2013;8(4):e61217. doi:10.1371/journal.pone.0061217. 23630581 PMC3632530

[77] Torgerson WS. Theory and methods of scaling. New York: Wiley; 1958.

[78] Bray JR, Curtis JT. An ordination of the upland forest communities of Southern Wisconsin. Ecol Monogr. 1957;27(4):325–349. doi:10.2307/1942268.

[79] Anders S, Huber W. Differential expression analysis for sequence count data. Genome Biol. 2010;11(10):R106. doi:10.1186/gb-2010-11-10-r106. 20979621 PMC3218662

[80] Mallick H, Chatterjee S, Chowdhury S, Chatterjee S, Rahnavard A, Hicks SC. Differential expression of single-cell RNA-seq data using Tweedie models. Stat Med. 2022;41(18):3492–3510. doi:10.1002/sim.9430. 35656596 PMC9288986

[81] Hea M. Tweedieverse - a unified statistical framework for differential analysis of multi-omics data. R package. 2021.

[82] Team RC. R: a language and environment for statistical computing. Austria: R Foundation for Statistical Computing Vienna; 2020.

[83] Brukner I, Longtin Y, Oughton M, Forgetta V, Dascal A. Assay for estimating total bacterial load: relative qPCR normalisation of bacterial load with associated clinical implications. Diagn Microbiol Infect Dis. 2015;83(1):1–6. doi:10.1016/j.diagmicrobio.2015.04.005. 26008123

