# Supplementary material for: Dietary resistant starch supplementation increases gut luminal deoxycholic acid abundance in mice
Source: Gut Microbes. 2024 Feb 20;16(1):2315632. doi: 10.1080/19490976.2024.2315632 (PMC10880513; doi:10.1080/19490976.2024.2315632)
Supplement: Supplement.docx [file KGMI_A_2315632_SM9682.docx]

**B C**


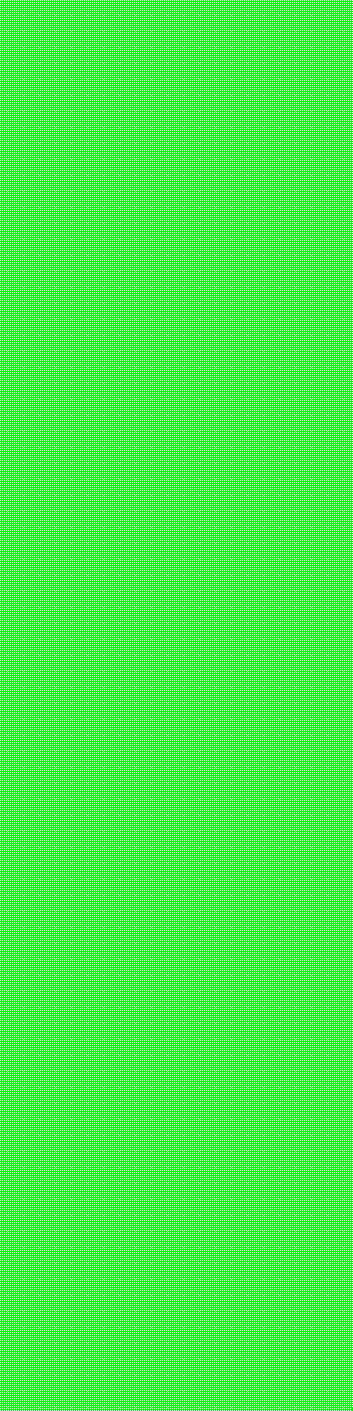

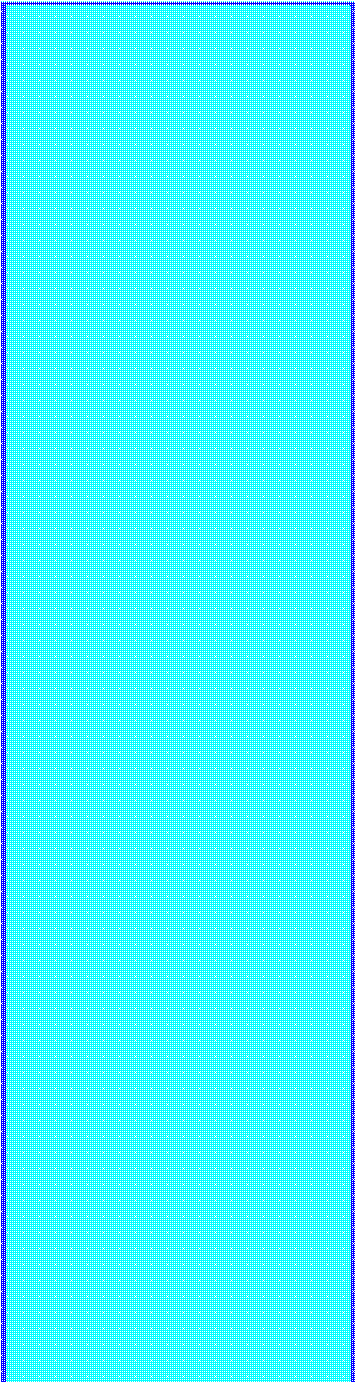

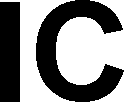

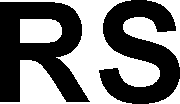

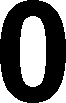

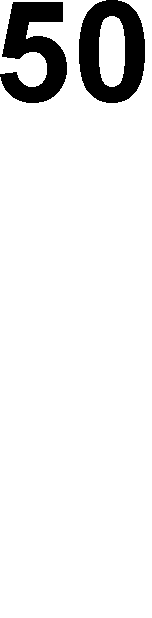

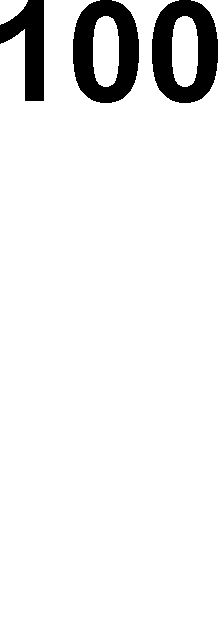

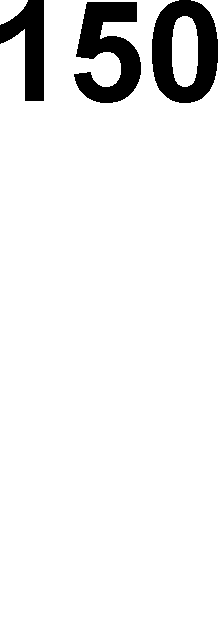

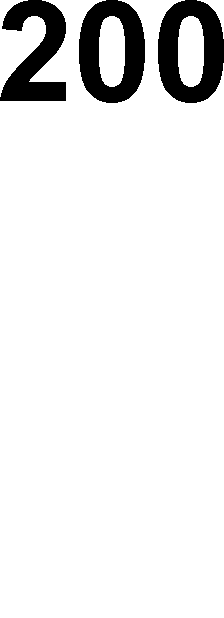

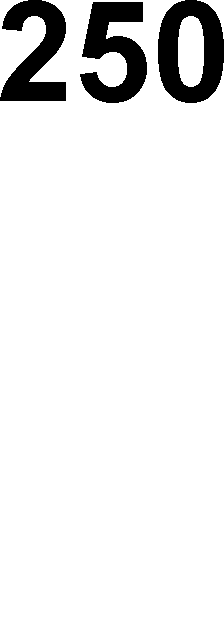

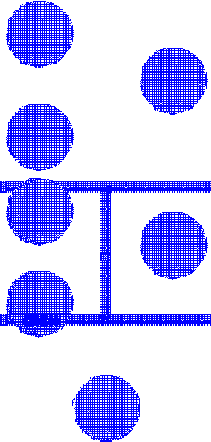

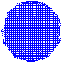

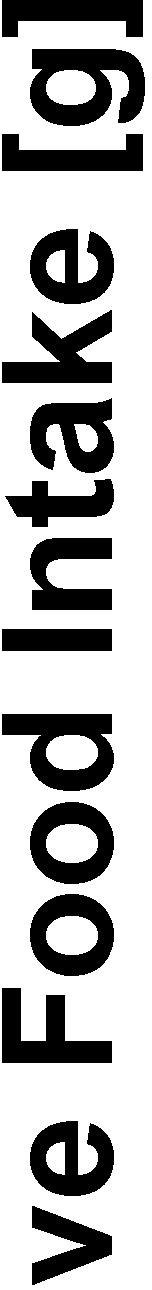

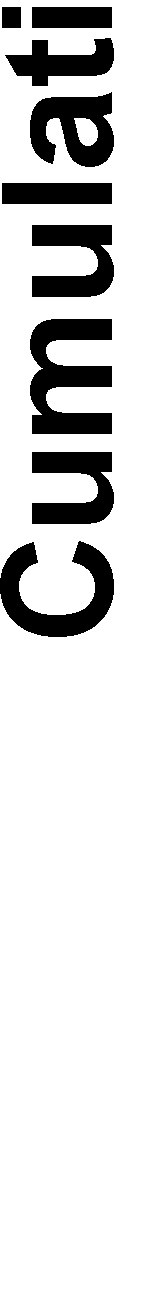

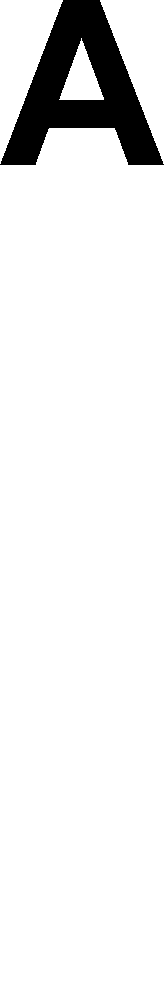

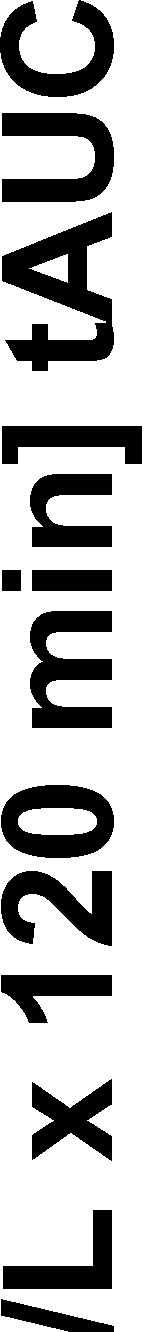

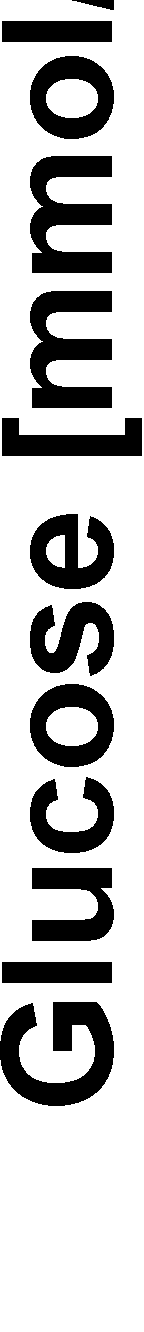


**40 25**


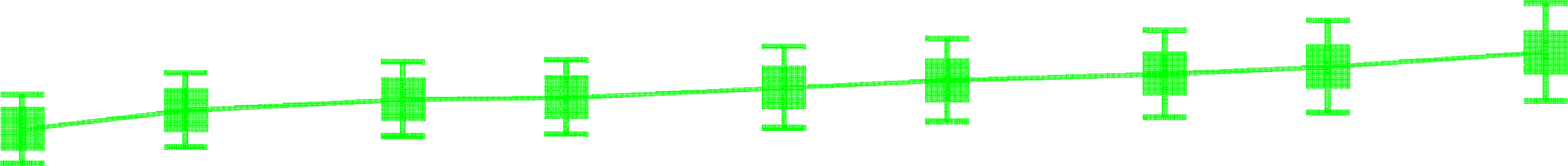

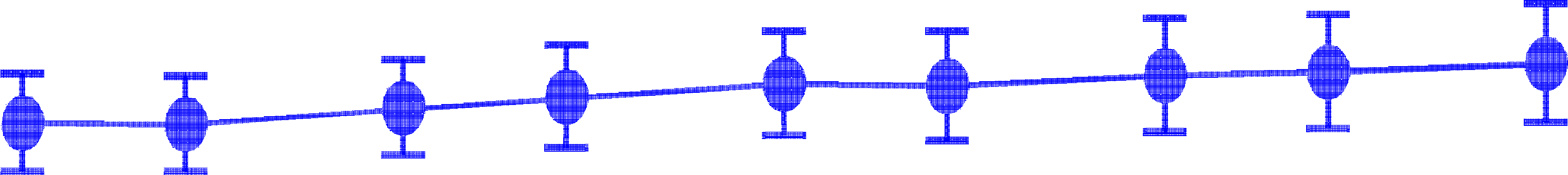

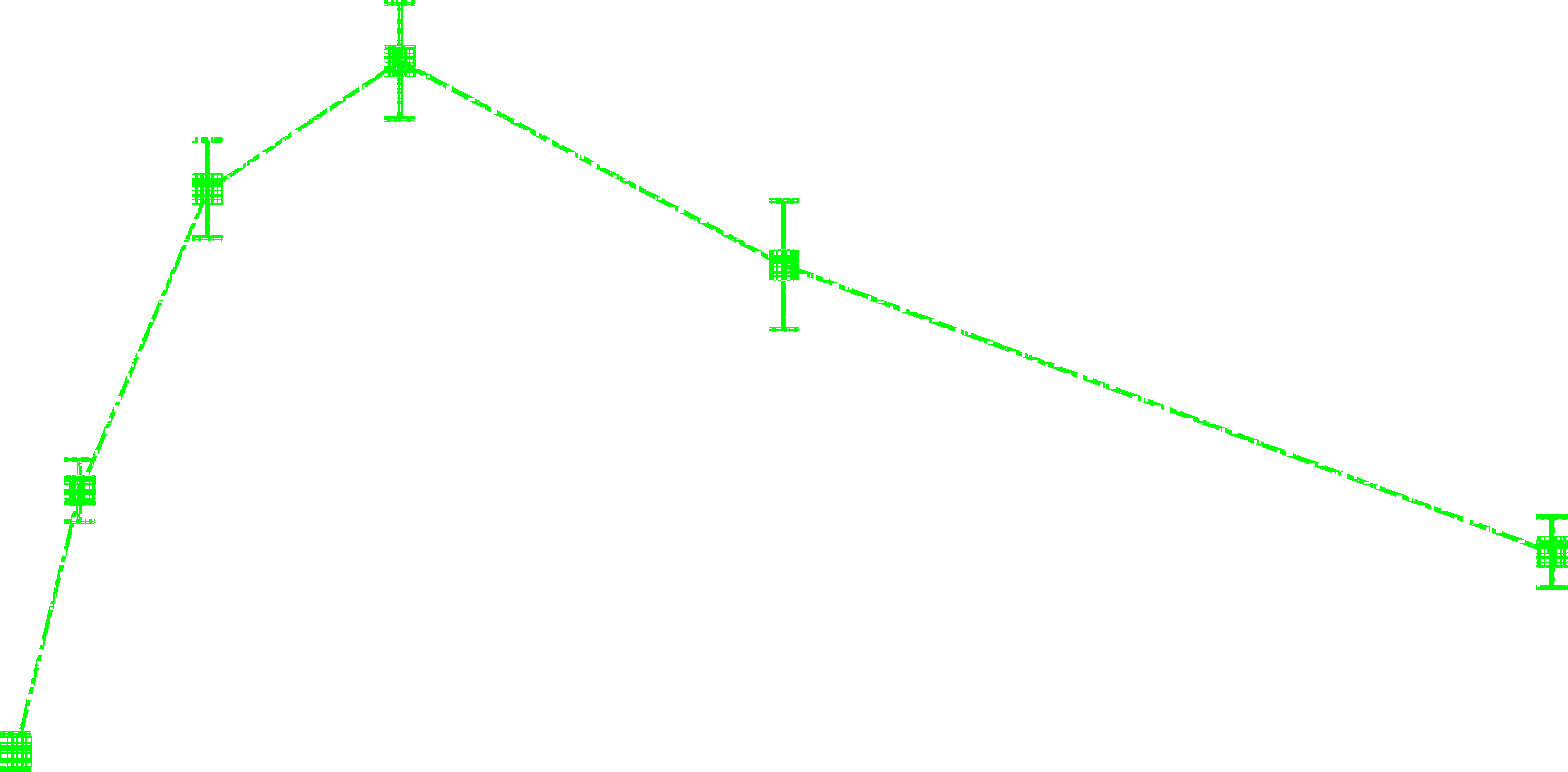

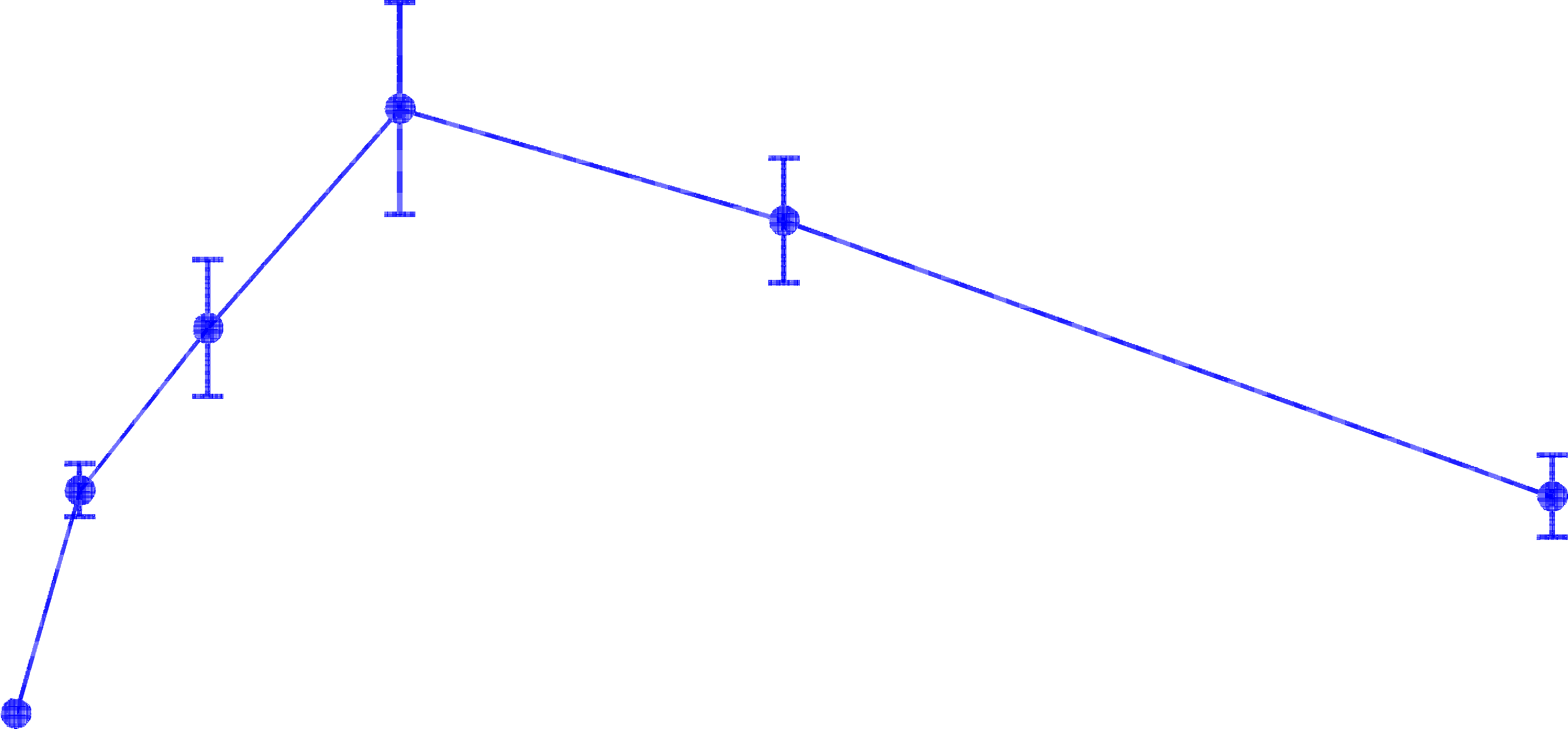

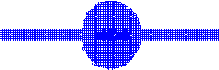

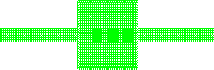


IC

RS


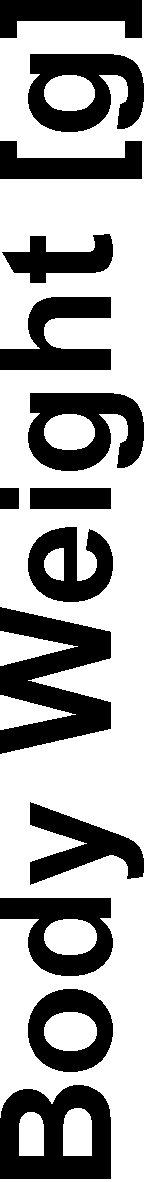
**30 20**

**Glucose [mmol/L]**

**15**

**20**

**10**

**10 5**

**0**

**0 10 20 30**

**Days of Treatment**

**0**

**0 5 15 30 60 120**

**Time (min)**

**E F**

**5 2.0**


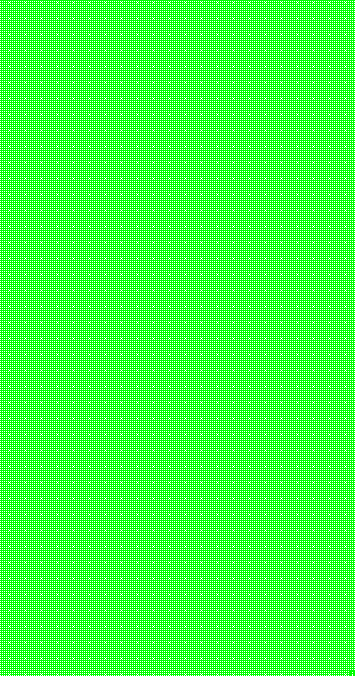

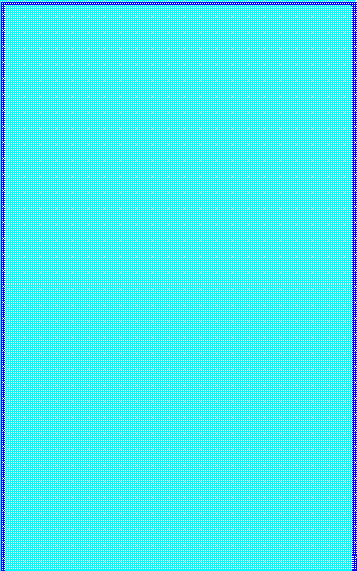

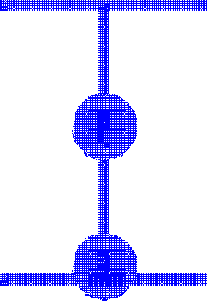

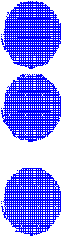

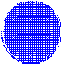

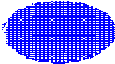

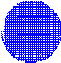

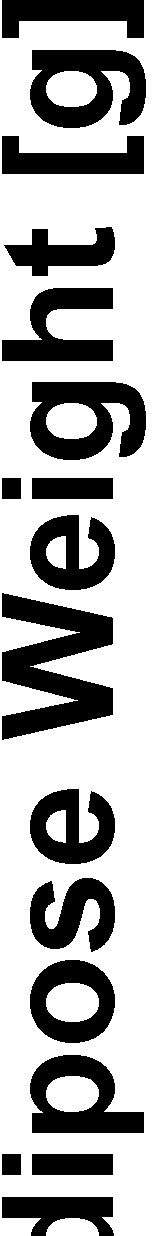

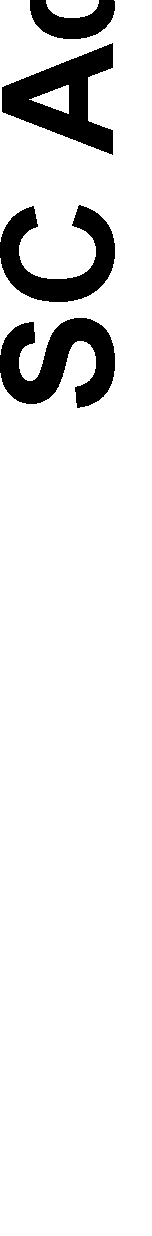

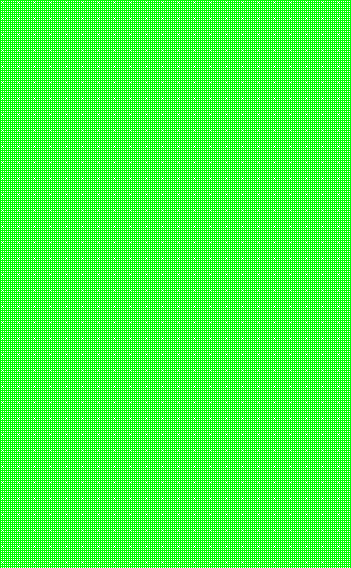

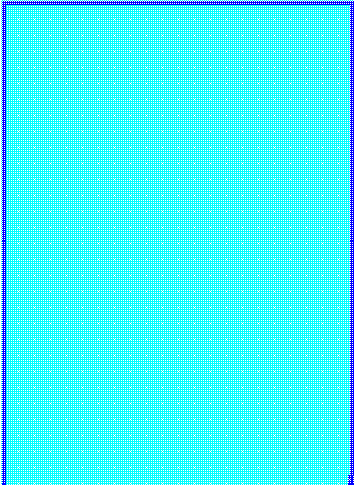

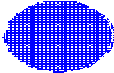

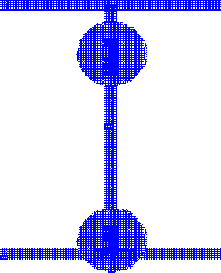

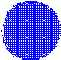

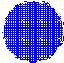

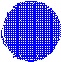

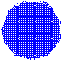

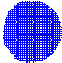


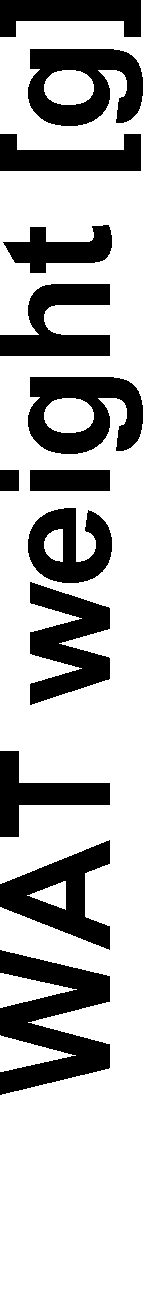
**4**

**1.5**

**3**

**1.0**

**2**

**1 0.5**

**G**

**2.0**


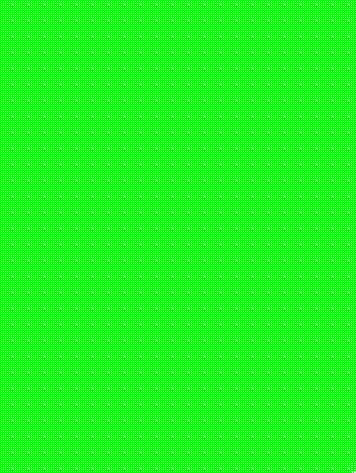

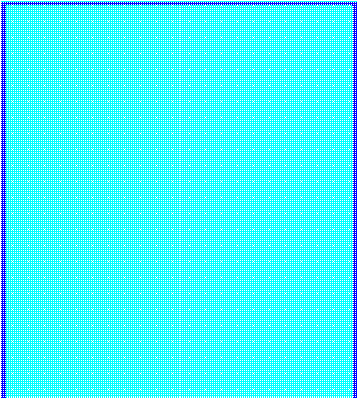

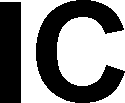

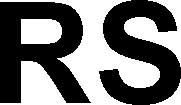

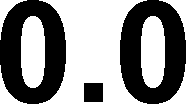

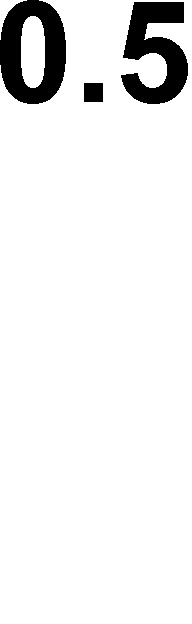

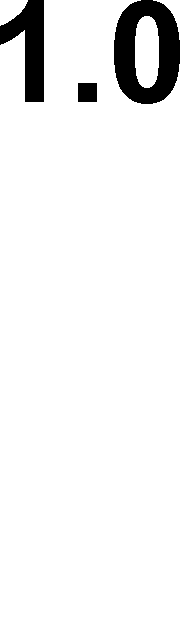

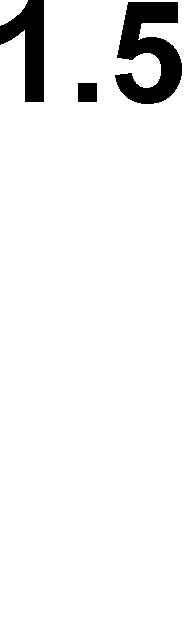

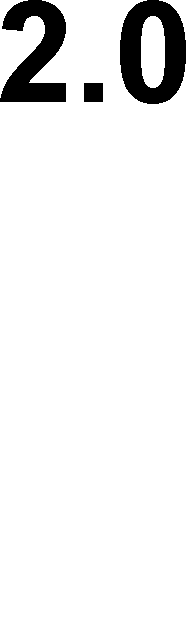

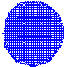

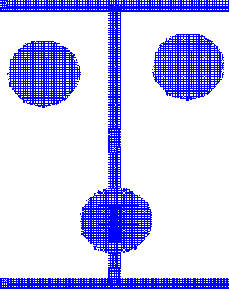

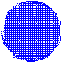

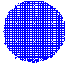

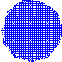

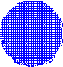

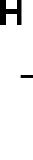

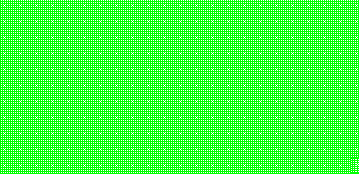

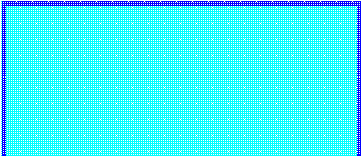

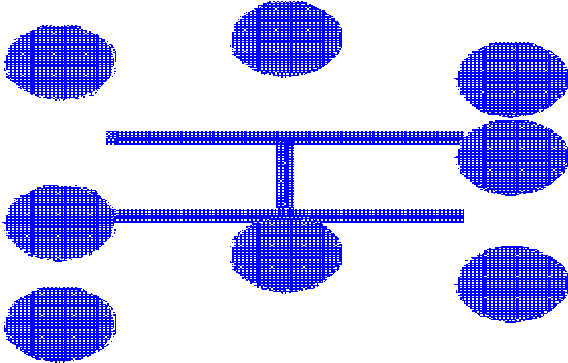

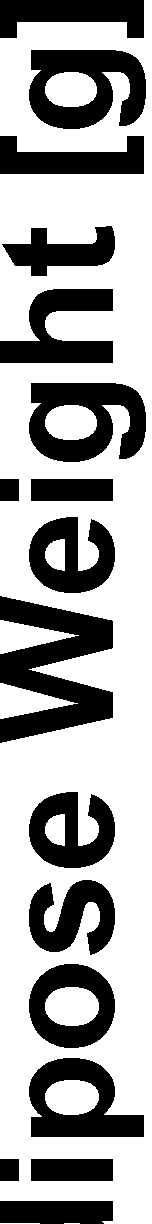

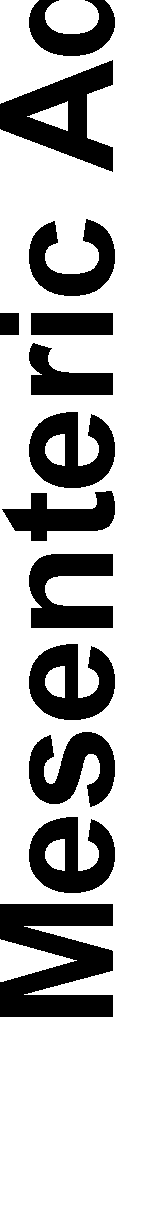

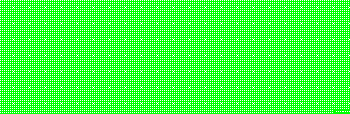

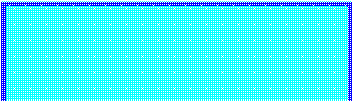

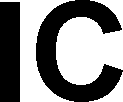

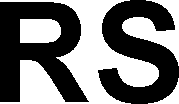

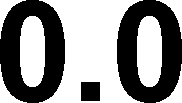

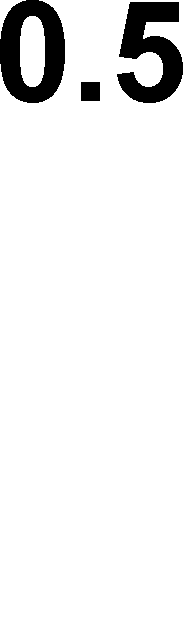

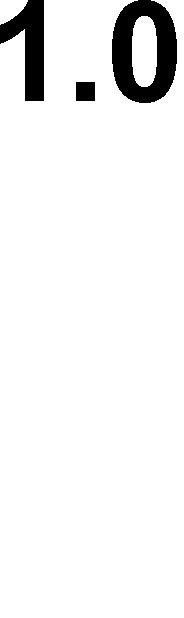

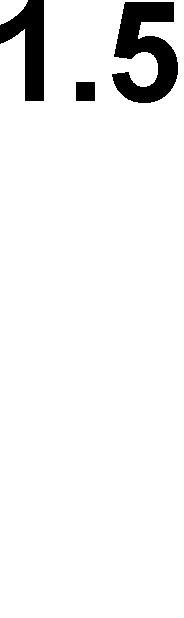

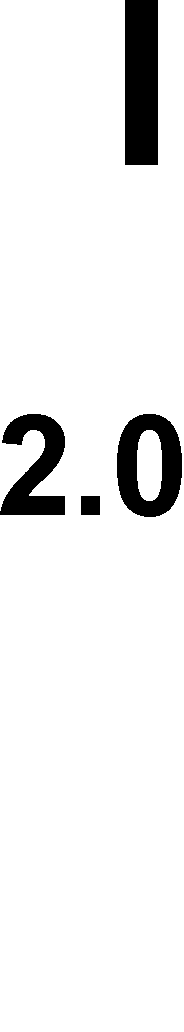

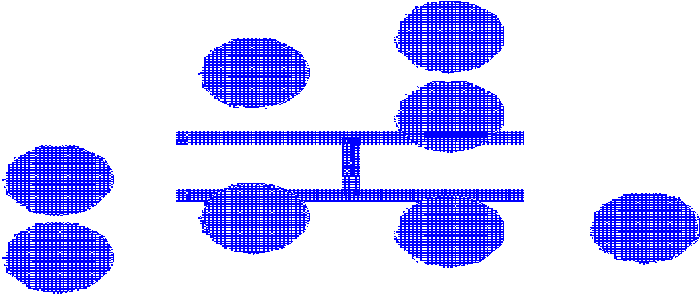

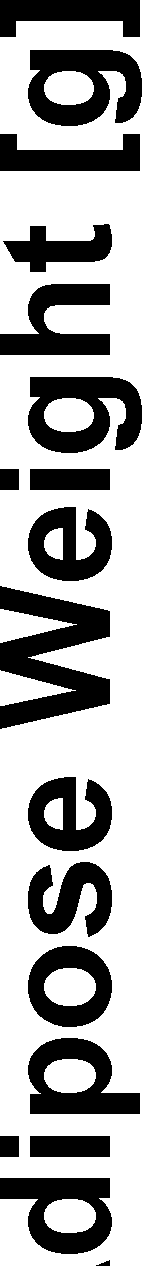

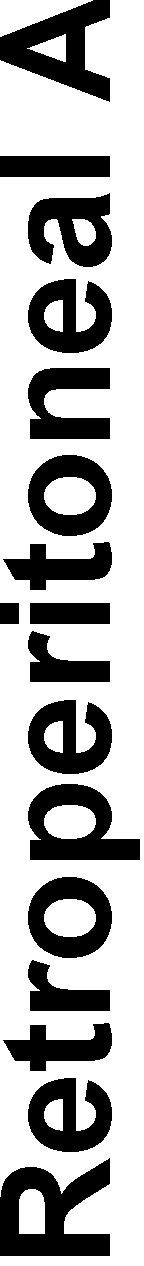


**Perigonadal Adipose Weight [g]**

**1.5**

**1.0**

**0.5**

**0**


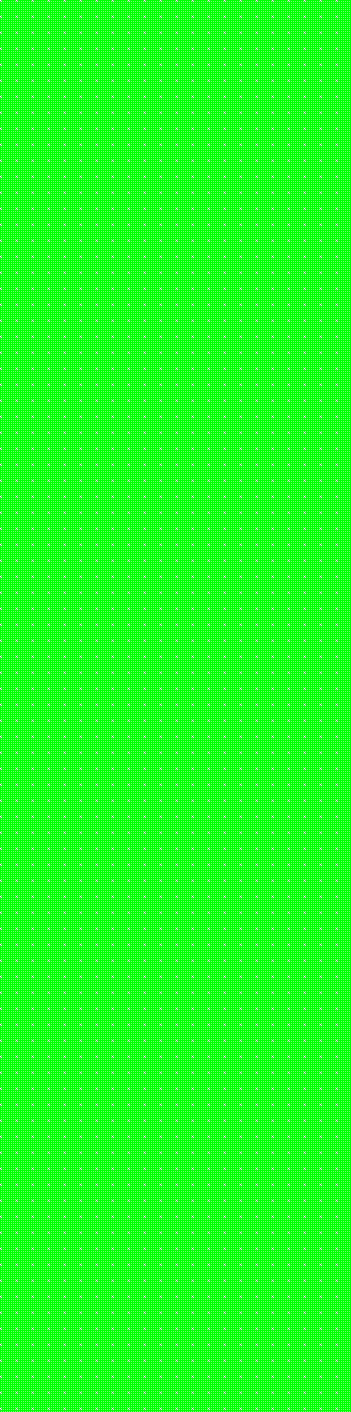

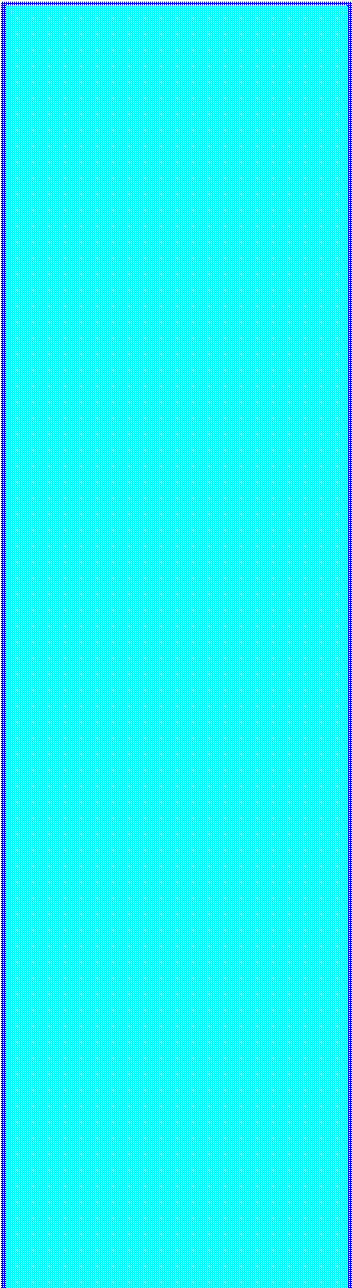

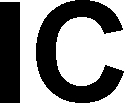

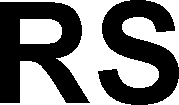

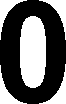

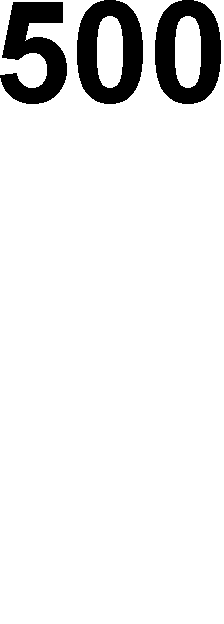

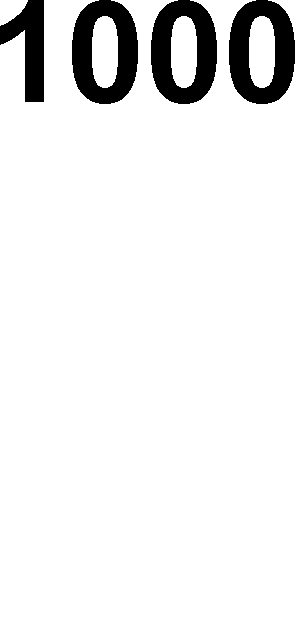

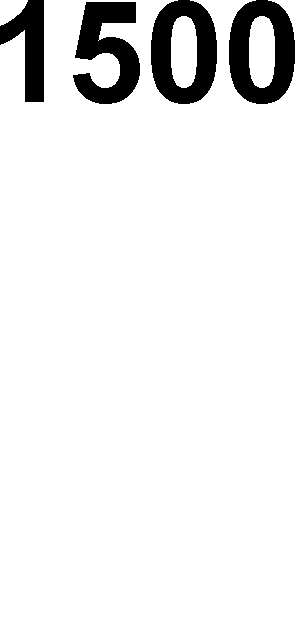

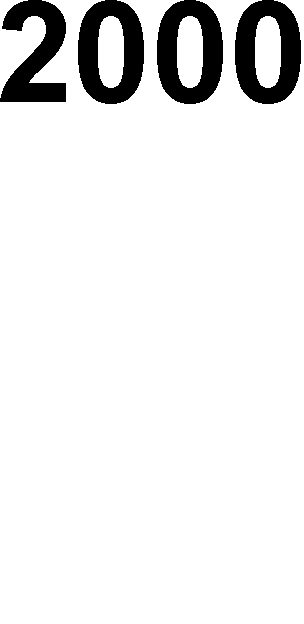

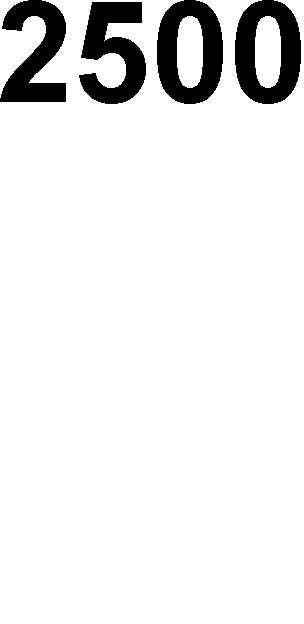

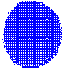

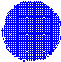

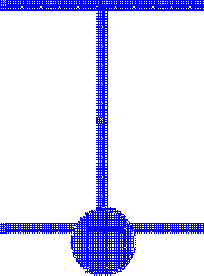

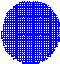

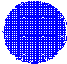

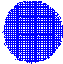

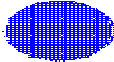

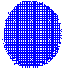

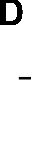


**IC RS**

**0.0**

**IC RS**

**0.0**

**IC RS**

# B C D


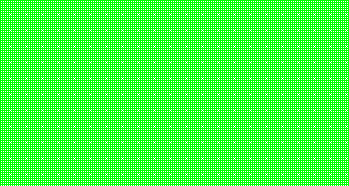

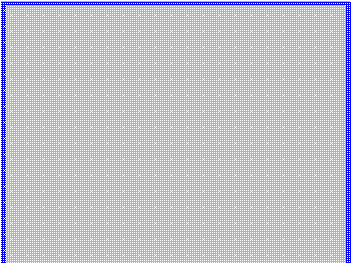

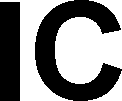

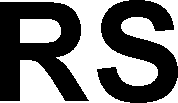

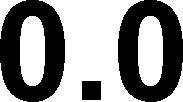


**1.0**

**Conjugated BA/Total BA**

**1.0**

**1.0**

**0.8**

**0.8**

**0.8**

**0.6**

**0.6**

**0.6**

**0.4**

**0.4**

**0.4**

**0.2**

**0.2**

**0.2**

**0.0**

**IC RS**

**0.0**

**IC RS**

**0.0**

**IC RS**

**E**

**1.0**

**F**

**1.0**

**G**

**0.10**

**H**

**0.010**

✱

**I**

**1.0**

**0.8**

**0.6**

**0.4**

**0.2**

**0.8**

**0.6**

**0.4**

**0.2**

**0.08**

**0.06**

**0.04**

**0.02**

**0.008**

**0.006**

**0.004**

**0.002**

**0.8**

**0.6**

**Total MCA/Total BA**

**0.4**

**0.2**

**0.0**

**IC RS**

**0.0**

**IC RS**

**0.00**

**IC RS**

**0.000**

**IC RS**

**0.0**

**IC RS**

# A

**1.0**

✱

**B**

✱

**1.0**

**C**

**1.0**

**D**

**1.0**

**0.8**

**0.8**

**0.8**

**0.8**

**0.6**

**0.6**

**0.6**

**0.6**

**0.4**

**0.4**

**0.4**

**0.4**

**0.2**

**0.2**

**0.2**

**0.2**

**0.0**

**IC RS**

**0.0**

**IC RS**

**0.0**

**IC RS**

**0.0**

**IC RS**

# E

**1.0**

**0.8**

**0.6**

**0.4**

**0.2**

**0.0**

**IC RS**

**F**

**1.0**

✱✱✱✱

**0.8**

**0.6**

**0.4**

**0.2**

**0.0**

**IC RS**

**G**

**0.10**

**0.08**

**0.06**

**0.04**

**0.02**

**0.00**

**IC RS**

**H**

**0.10**

**0.08**

**0.06**

**0.04**

**0.02**

**0.00**

**IC RS**

**I**

**1.0**

✱✱✱

**0.8**

**0.6**

**0.4**

**0.2**

**0.0**

**IC RS**

# A B

**4 4**

**3 3**

**Liver *Cyp2c70* [AU]**

**2 2**

**1 1**

**0**

**IC RS**

**0**

**IC RS**

# D

✱

**4**

**3**

**Ileal *OSTα* [AU]**

**Ileal *OSTβ* [AU]**

**2**

**1**

**0**

**IC RS**

**Supplemental Table S1. Gut luminal bile acid concentrations**

|  | **Gut luminal bile acid concentrations (nmol/g) after one month of diet intervention** | | |
| --- | --- | --- | --- |
|  | **IC** | **RS** | **p-value** |
| **CA** | 223.83 ± 99.11 | 16.95 ± 3.22 | 0.03 |
| **CDCA** | 1.50 ± 0.68 | 0.00 ± 0.00 | 0.03 |
| **DCA** | 258.90 ± 67.88 | 61.47 ± 16.27 | <0.01 |
| **GCA** | 0.28 ± 0.21 | 0.00 ± 0.00 | 0.14 |
| **GDCA** | 0.43 ± 0.13 | 0.27 ± 0.08 | 0.28 |
| **GUDCA** | 0.11 ± 0.05 | 0.00 ± 0.00 | 0.04 |
| **HDCA** | 18.72 ± 6.94 | 0.82 ± 0.32 | 0.01 |
| **LCA** | 5.17 ± 1.79 | 1.51 ± 0.49 | 0.04 |
| **α-MCA** | 613.52 ± 228.37 | 18.74 ± 3.72 | 0.01 |
| **β-MCA** | 429.33 ± 167.13 | 70.00 ± 14.54 | 0.03 |
| **TCA** | 54.22 ± 39.90 | 1.98 ± 0.58 | 0.16 |
| **TCDCA** | 2.94 ± 2.63 | 0.02 ± 0.02 | 0.23 |
| **TDCA** | 3.81 ± 1.47 | 0.30 ± 0.12 | 0.02 |
| **TLCA** | 0.16 ± 0.08 | 0.00 ± 0.00 | 0.04 |
| **TMCA (α+β)** | 238.17 ± 125.73 | 14.63 ± 2.77 | 0.06 |
| **TUDCA** | 5.15 ± 2.44 | 0.30 ± 0.09 | 0.04 |
| **UDCA** | 14.32 ± 5.19 | 2.36 ± 0.48 | 0.02 |
| **Total** | 1870.56 ± 556.30 | 189.34 ± 35.76 | <0.01 |
|  | **Gut luminal bile acid concentrations (nmol/g) after two months of diet intervention** | | |
|  | **IC** | **RS** | **p-value** |
| **CA** | 667.91 ± 200.66 | 236.82 ± 100.84 | 0.08 |
| **CDCA** | 6.61 ± 2.60 | 1.03 ± 0.65 | 0.06 |
| **DCA** | 446.38 ± 145.30 | 866.47 ± 492.94 | 0.43 |
| **GCA** | 0.86 ± 0.35 | 0.16 ± 0.08 | 0.07 |
| **GDCA** | 0.52 ± 0.14 | 0.74 ± 0.17 | 0.34 |
| **GUDCA** | 0.15 ± 0.06 | 0.00 ± 0.00 | 0.02 |
| **HDCA** | 29.31 ± 5.64 | 8.67 ± 6.16 | 0.03 |
| **LCA** | 5.89 ± 1.43 | 5.27 ± 2.55 | 0.83 |
| **α-MCA** | 600.34 ± 122.99 | 35.63 ± 9.76 | <0.001 |
| **β-MCA** | 858.81 ± 243.72 | 127.79 ± 22.94 | <0.01 |
| **TCA** | 126.16 ± 106.32 | 1.86 ± 1.35 | 0.26 |
| **TCDCA** | 4.12 ± 3.08 | 0.03 ± 0.03 | 0.21 |
| **TDCA** | 7.28 ± 3.25 | 2.42 ± 1.24 | 0.18 |
| **TMCA (α+β)** | 199.89 ± 127.44 | 1.49 ± 0.70 | 0.14 |
| **TUDCA** | 3.70 ± 2.29 | 0.00 ± 0.00 | 0.13 |
| **UDCA** | 28.99 ± 5.69 | 3.34 ± 0.83 | <0.001 |
| **Total** | 2986.92 ± 1291.73 | 1291.73 ± 574.17 | 0.07 |

|  | **Gut luminal bile acids normalized to total bile acids after one month of diet intervention (proportion of total)** | | |
| --- | --- | --- | --- |
|  | **IC** | **RS** | **p-value** |
| **CA** | 0.09635 ± 0.02142 | 0.09588 ± 0.01504 | 0.99 |
| **CDCA** | 0.00056 ± 0.00023 | 0.00000 ± 0.00000 | 0.01 |
| **DCA** | 0.18084 ± 0.04389 | 0.2166 ± 0.03270 | 0.08 |
| **GCA** | 0.00009 ± 0.00007 | 0.00000 ± 0.00000 | 0.16 |
| **GDCA** | 0.00029 ± 0.00013 | 0.00137 ± 0.00049 | 0.07 |
| **GUDCA** | 0.00003 ± 0.00002 | 0.00000 ± 0.00000 | 0.04 |
| **HDCA** | 0.00811 ± 0.00226 | 0.00384 ± 0.00164 | 0.14 |
| **LCA** | 0.00463 ± 0.00163 | 0.00694 ± 0.00141 | 0.30 |
| **α-MCA** | 0.32606 ± 0.05504 | 0.13408 ± 0.03976 | 0.01 |
| **β-MCA** | 0.23339 ± 0.03095 | 0.36202 ± 0.02415 | <0.01 |
| **TCA** | 0.02195 ± 0.01292 | 0.01114 ± 0.00377 | 0.39 |
| **TCDCA** | 0.00129 ± 0.00088 | 0.00022 ± 0.00022 | 0.21 |
| **TDCA** | 0.00192 ± 0.00060 | 0.00102 ± 0.00036 | 0.19 |
| **TLCA** | 0.00005 ± 0.00003 | 0.00000 ± 0.00000 | 0.05 |
| **TMCA (α+β)** | 0.11512 ± 0.03910 | 0.08620 ± 0.02210 | 0.51 |
| **TUDCA** | 0.00209 ± 0.00072 | 0.00156 ± 0.00062 | 0.58 |
| **UDCA** | 0.00722 ± 0.00134 | 0.01408 ± 0.00308 | 0.08 |
|  | **Gut luminal bile acids normalized to total bile acids after two months of diet intervention (proportion of total)** | | |
|  | **IC** | **RS** | **p-value** |
| **CA** | 0.20599 ± 0.02226 | 0.21905 ± 0.06737 | 0.86 |
| **CDCA** | 0.00317 ± 0.00130 | 0.00271 ± 0.00243 | 0.87 |
| **DCA** | 0.16061 ± 0.04475 | 0.54838 ± 0.08415 | <0.01 |
| **GCA** | 0.00023 ± 0.00009 | 0.00011 ± 0.00007 | 0.32 |
| **GDCA** | 0.00026 ± 0.00007 | 0.00128 ± 0.00047 | 0.05 |
| **GUDCA** | 0.00005 ± 0.00002 | 0.00000 ± 0.00000 | 0.03 |
| **HDCA** | 0.01328 ± 0.00337 | 0.00326 ± 0.00142 | 0.02 |
| **LCA** | 0.00279 ± 0.00064 | 0.00685 ± 0.00350 | 0.27 |
| **α-MCA** | 0.24352 ± 0.04886 | 0.04247 ± 0.00793 | <0.01 |
| **β-MCA** | 0.28444 ± 0.03805 | 0.16653 ± 0.02489 | 0.02 |
| **TCA** | 0.02370 ± 0.01636 | 0.00151 ± 0.00081 | 0.20 |
| **TCDCA** | 0.00086 ± 0.00050 | 0.00002 ± 0.00002 | 0.11 |
| **TDCA** | 0.00187 ± 0.00054 | 0.00139 ± 0.00029 | 0.45 |
| **TMCA (α+β)** | 0.04659 ± 0.01985 | 0.00151 ± 0.00048 | 0.04 |
| **TUDCA** | 0.00094 ± 0.00053 | 0.00000 ± 0.00000 | 0.10 |
| **UDCA** | 0.01169 ± 0.00234 | 0.00493 ± 0.00176 | 0.04 |

|  | **Circulating bile acid concentrations (nM) after one month of diet intervention** | | |
| --- | --- | --- | --- |
|  | **IC** | **RS** | **p-value** |
| **CA** | 4159.85 ± 906.10 | 3937.65 ± 1044.74 | 0.95 |
| **CDCA** | 134.45 ± 93.28 | 143.74 ± 61.66 | 0.93 |
| **DCA** | 580.37 ± 175.70 | 740.88 ± 230.95 | 0.60 |
| **GCA** | 9.61 ± 5.83 | 8.30 ± 1.99 | 0.82 |
| **GCDCA** | 0.71 ± 0.71 | 0.00 ± 0.00 | 0.28 |
| **GDCA** | 1.48 ± 0.74 | 0.00 ± 0.00 | 0.04 |
| **GUDCA** | 0.46 ± 0.46 | 0.00 ± 0.00 | 0.28 |
| **HDCA** | 55.41 ± 27.48 | 4.56 ± 2.12 | 0.05 |
| **LCA** | 27.30 ± 7.59 | 41.08 ± 14.04 | 0.43 |
| **α-MCA** | 1475.17 ± 906.10 | 174.19 ± 79.52 | 0.13 |
| **β-MCA** | 3276.74 ± 2521.47 | 899.79 ± 374.51 | 0.31 |
| **TCA** | 1494.99 ± 1129.65 | 909.65 ± 232.41 | 0.58 |
| **TCDCA** | 249.50 ± 215.97 | 31.89 ± 7.82 | 0.27 |
| **TDCA** | 59.28 ± 26.59 | 46.21 ± 17.03 | 0.67 |
| **TLCA** | 2.34 ± 1.38 | 1.38 ± 0.50 | 0.49 |
| **TMCA (α+β)** | 4642.37 ± 4243.68 | 172.73 ± 33.30 | 0.25 |
| **TUDCA** | 170.21 ± 62.74 | 33.98 ± 3.69 | 0.03 |
| **UDCA** | 350.28 ± 81.27 | 497.93 ± 230.60 | 0.59 |
| **Total** | 16690.53 ± 12526.39 | 7643.95 ± 1970.38 | 0.44 |
|  | **Circulating bile acid concentrations (nM) after two months of diet intervention** | | |
|  | **IC** | **RS** | **p-value** |
| **CA** | 284.27 ± 119.88 | 400.24 ± 97.65 | 0.47 |
| **CDCA** | 11.98 ± 6.80 | 17.41 ± 8.81 | 0.63 |
| **DCA** | 375.24 ± 137.24 | 367.74 ± 117.95 | 0.97 |
| **GCA** | 2.52 ± 0.77 | 2.92 ± 0.96 | 0.75 |
| **GCDCA** | 0.00 ± 0.00 | 0.36 ± 0.36 | 0.33 |
| **GDCA** | 0.68 ± 0.68 | 0.36 ± 0.36 | 0.69 |
| **HDCA** | 32.69 ± 12.86 | 4.97 ± 3.98 | 0.06 |
| **LCA** | 12.50 ± 1.98 | 7.61 ± 2.02 | 0.11 |
| **α-MCA** | 226.54 ± 92.31 | 10.40 ± 2.92 | 0.03 |
| **β-MCA** | 480.70 ± 240.88 | 94.90 ± 32.02 | 0.13 |
| **TCA** | 628.59 ± 313.76 | 573.19 ± 209.09 | 0.89 |
| **TCDCA** | 36.66 ± 8.91 | 14.43 ± 3.99 | 0.04 |
| **TDCA** | 140.96 ± 46.19 | 67.58 ± 23.44 | 0.18 |
| **TLCA** | 0.95 ± 0.47 | 0.00 ± 0.00 | 0.06 |
| **TMCA (α+β)** | 399.97 ± 186.62 | 44.88 ± 17.00 | 0.08 |
| **TUDCA** | 124.24 ± 27.71 | 14.82 ± 3.27 | <0.01 |
| **UDCA** | 84.73 ± 38.28 | 30.45 ± 8.32 | 0.19 |
| **Total** | 2843.21 ± 995.16 | 1652.27 ± 289.50 | 0.27 |

|  | **Circulating bile acids normalized to total bile acids after one month of diet intervention (proportion of total)** | | |
| --- | --- | --- | --- |
|  | **IC** | **RS** | **p-value** |
| **CA** | 0.20678 ± 0.01657 | 0.52263 ± 0.01496 | <0.00001 |
| **CDCA** | 0.00971 ± 0.00303 | 0.01848 ± 0.00332 | 0.08 |
| **DCA** | 0.11912 ± 0.02953 | 0.09919 ± 0.02135 | 0.58 |
| **GCA** | 0.00110 ± 0.00040 | 0.00133 ± 0.00035 | 0.67 |
| **GCDCA** | 0.00001 ± 0.00001 | 0.00000 ± 0.00000 | 0.28 |
| **GDCA** | 0.00046 ± 0.00027 | 0.00000 ± 0.00000 | 0.08 |
| **HDCA** | 0.01067 ± 0.00385 | 0.00058 ± 0.00027 | <0.01 |
| **LCA** | 0.00464 ± 0.00103 | 0.00491 ± 0.00090 | 0.85 |
| **α-MCA** | 0.11976 ± 0.01907 | 0.02233 ± 0.00370 | <0.0001 |
| **β-MCA** | 0.17104 ± 0.02253 | 0.10203 ± 0.01051 | <0.01 |
| **TCA** | 0.10589 ± 0.02736 | 0.12545 ± 0.01686 | 0.53 |
| **TCDCA** | 0.01011 ± 0.00233 | 0.00468 ± 0.00075 | 0.03 |
| **TDCA** | 0.01492 ± 0.00627 | 0.00613 ± 0.00140 | 0.15 |
| **TLCA** | 0.00021 ± 0.00010 | 0.00015 ± 0.00006 | 0.56 |
| **TMCA (α+β)** | 0.13622 ± 0.03143 | 0.02679 ± 0.00413 | <0.01 |
| **TUDCA** | 0.02646 ± 0.00490 | 0.00608 ± 0.00085 | <0.001 |
| **UDCA** | 0.0629 ± 0.01310 | 0.05924 ± 0.01301 | 0.85 |
|  | **Circulating bile acids normalized to total bile acids after two months of diet intervention (proportion of total)** | | |
|  | **IC** | **RS** | **p-value** |
| **CA** | 0.12996 ± 0.03498 | 0.26734 ± 0.06346 | 0.08 |
| **CDCA** | 0.00483 ± 0.00225 | 0.01393 ± 0.00776 | 0.28 |
| **DCA** | 0.13813 ± 0.03206 | 0.21772 ± 0.06272 | 0.28 |
| **GCA** | 0.00084 ± 0.00030 | 0.00160 ± 0.00051 | 0.22 |
| **GCDCA** | 0.00000 ± 0.00000 | 0.00020 ± 0.00020 | 0.33 |
| **GDCA** | 0.00008 ± 0.00008 | 0.00040 ± 0.00040 | 0.44 |
| **HDCA** | 0.01013 ± 0.00280 | 0.00250 ± 0.00217 | 0.05 |
| **LCA** | 0.00747 ± 0.00182 | 0.00465 ± 0.00107 | 0.20 |
| **α-MCA** | 0.07759 ± 0.01481 | 0.00661 ± 0.00163 | <0.001 |
| **β-MCA** | 0.11846 ± 0.03063 | 0.05454 ± 0.01546 | 0.08 |
| **TCA** | 0.20411 ± 0.04331 | 0.32759 ± 0.06566 | 0.14 |
| **TCDCA** | 0.01759 ± 0.00360 | 0.00950 ± 0.00219 | 0.08 |
| **TDCA** | 0.05848 ± 0.01427 | 0.03788 ± 0.01058 | 0.27 |
| **TLCA** | 0.00020 ± 0.00011 | 0.00000 ± 0.00000 | 0.08 |
| **TMCA (α+β)** | 0.14329 ± 0.02653 | 0.02424 ± 0.00433 | <0.001 |
| **TUDCA** | 0.06066 ± 0.01118 | 0.00997 ± 0.00210 | <0.001 |
| **UDCA** | 0.02818 ± 0.00841 | 0.02135 ± 0.00797 | 0.56 |

|  | **g** | |
| --- | --- | --- |
|  | **IC** | **RS** |
| **Amoica** | 500 | 100 |
| **High Maize 260®** | 0 | 500 |
| **Sucrose** | 30 | 30 |
| **Casein** | 140 | 140 |
| **Cellulose** | 100 | 0 |
| **Fat** | 181.9 | 181.9 |
| **Mineral Mix** | 35 | 35 |
| **Vitamin Mix** | 10 | 10 |
| **Choline Bitartrate** | 2.36 | 2.36 |
| **L-Cystine** | 1.8 | 1.8 |

**Supplemental Table S6. Primer Sequences.**

| **Primer** | **Forward 5’** 🡪 **3’** | **Reverse 5’** 🡪 **3’** |
| --- | --- | --- |
| *Cyp 7a1* | ACGCACCTCGTGATCCTCTGGG | GGCTGCTTTCATTGCTTCAGGGCT |
| *Cyp8b1* | ATCGCCTGAAGCCCGTGCAG | AGCTGGGGAGAGGAAGGAGTGC |
| *Cyp2c70* | TCCCAAGGGCACAAGTGTAAT | GCTGTAAGATGTTGGTCAGGATT |
| *Asbt* | GGGGTATCTTCGTGGGCTTC | TGCTAACACTGAGGTCCATGTC |
| *Ostα* | GGCATCTATGACCCAGGAGA | TGGATCCCATGTTCTGTTCA |
| *Ostβ* | GACCACAGTGCAGAGAAAGC | ATTCCAAGGAGCCGCATCT |
| *Actin* | CAACGAGCGGTTCCGAT | GCCACAGGATTCCATACCCA |
| *Total bacterial number* | AATAAATCATAAACTCCTAC- GGGAGGCAGCAGT | AATAAATCATAACCTAGC- TATTACCGCGGCTGCT |

**Supplemental Figures Legends**

**Supplemental Figure 1. Measures of metabolic health were unchanged after one month of RS supplementation. A**) Cumulative food intake (days 1-25 of RS or IC intervention) and **B**) body weight in mice receiving IC or RS diet for 1 month. **C**) Glucose concentrations and, **D**) the total area under the curve during oral glucose tolerance testing in mice receiving IC or RS. **E**) Total white adipose weight, **F**) subcutaneous adipose tissue weight, **G**) mesenteric adipose tissue weight, **H**) perigonadal adipose tissue weight, and **I**) retroperitoneal adipose tissue weight in mice receiving IC or RS diet for 1 month. Data presented as mean ± SEM, *n*=8 per group.

**Supplemental Figure 2. One month of RS supplementation altered gut luminal bile acid profile. A**) Conjugated bile acids, **B**) unconjugated bile acids, **C**) primary bile acids, **D**) secondary bile acids, **E**) DCA, **F**) CA, **G**), LCA, **H**) CDCA, **I**) and total MCA levels expressed as a proportion of total bile acids in cecal contents collected from mice receiving IC or RS diet for 1 month. Data presented as mean ± SEM, *n*=8 per group. **P*<0.05 by Student’s t-test.

**serum after one month of RS supplementation. A**)Conjugated bile acids, **B**) Unconjugated bile acids, **C**) primary bile acids, **D**) secondary bile acids, **E**) DCA, **F**) CA, **G**) LCA, **H**) CDCA, and **I**) total MCA levels expressed as a proportion of total bile acids in fasted serum samples collected from mice receiving IC or RS diet for 1 month. Data presented as mean ± SEM, *n*=8 per group. **P*<0.05, ****P*<0.001 by Student's t-test.

**Supplemental Figure 4. mRNA expression of hepatic bile acid producing enzymes was not changed by one month of RS intervention, but gut bile acid transporter mRNA expression was upregulated. A**) *Cyp7a1*, **B**) *Cyp8b1,* and **C**) *Cyp2c70* mRNA expression in liver collected after 1 month of RS or IC feeding. **D**) *Asbt*, **E**) *Ostα*, and **F**) *Ostβ* mRNA expression in ileum collected after 1 month of RS or IC feeding. Data presented as mean ± SEM, *n*=8 per group.

**P*<0.05 by Student's t-test.
